# Analytical results for directional and quadratic selection gradients for log‐linear models of fitness functions

**DOI:** 10.1111/evo.14486

**Published:** 2022-05-18

**Authors:** Michael B. Morrissey, I. B. J. Goudie

**Affiliations:** ^1^ School of Biology University of St Andrews St Andrews Fife KY16 9TH UK; ^2^ School of Mathematics and Statistics University of St Andrews St Andrews Fife KY16 9SS UK

**Keywords:** Capture‐mark‐recapture, fitness, generalised linear model, natural selection, selection gradients, survival analysis

## Abstract

Log‐linear models are widely used for assessing determinants of fitness in empirical studies, for example, in determining how reproductive output depends on trait values or environmental conditions. Similarly, theoretical works of fitness and natural selection employ log‐linear models, often with a negative quadratic term, generating Gaussian fitness functions. However, in the specific application of regression‐based analysis of natural selection, such models are rarely employed. Rather, OLS regression is the predominant means of assessing the form of natural selection. OLS regressions allow specific evolutionary quantitative parameters, selection gradients, to be estimated, and benefit from the fact that the associated statistical models are easily applied. We examine whether selection gradients can be directly expressed in terms of the coefficients of models using exponential fitness functions with linear or quadratic arguments. Such models can be easily fitted with generalized linear models (GLMs). The expressions we obtain coincide with those for Gaussian functions, but relax the major constraint that the (log) fitness function is concave (downwardly curved). Additionally these results lead to univariate and multivariate analyses of both linear and quadratic selection that potentially incorporate pragmatic and interpretable models of fitness functions, where the parameters can be related analytically to selection gradients, and that can be operationalized using widely available statistical tools.

The characterization of natural selection, especially in the wild, has long been a major research theme in evolutionary ecology and evolutionary quantitative genetics (Endler 1986, Kingsolver et al. 2001, Lande & Arnold 1983, Manly 1985, Weldon 1901). In recent decades, regression‐based approaches have been used to obtain direct selection gradients (especially following Lande & Arnold 1983), which represent the direct effects of traits on fitness. These, and related, measures of selection have an explicit justification in quantitative genetic theory (Lande 1979, Lande & Arnold 1983), which provides the basis for comparison among traits, taxa, and so on, and ultimately allows meta‐analysis (e.g., Kingsolver et al. 2001). Selection gradients can characterize both directional selection and aspects of nonlinear selection, and so are a very powerful concept in evolutionary quantitative genetics.

The selection gradient may be defined as the vector of partial derivatives of relative fitness with respect to phenotype, averaged over the distribution of phenotype observed in a population. This definition is equivalent to other existing definitions when the phenotype follows a Gaussian distribution (Walsh & Lynch 2018), an assumption we rely on for most of our results. Following Lande & Arnold (1983), given an arbitrary function W(z) for expected fitness of a (multivariate) phenotype z, a general expression for the directional selection gradient vector β is

(1) $$\beta ={\bar{W}}^{-1}\int \frac{\partial W(z)}{\partial z^{\prime }}p\left(z\right)dz\;,$$

where p(z) is the probability density function of phenotype, with z being a column vector, and $\bar{W}$ is mean fitness. Mean fitness can itself be obtained by ∫W(z)p(z)dz. A quadratic selection gradient can also be defined as the average curvature (similarly standardised), rather than the average slope, of the relative fitness function,

(2) $$\gamma ={\bar{W}}^{-1}\int \frac{\partial^{2}W\left(z\right)}{\partial z\partial z^{\prime }}p\left(z\right)dz.$$

The directional selection gradient has a direct relationship to evolutionary change, assuming that breeding values (the additive genetic component of individual phenotype, Falconer 1960) are multivariate normally distributed, following the Lande (1979) equation

(3) $$\Delta \bar{z}=G\beta \;,$$

where $\Delta \bar{z}$ is per‐generation evolutionary change, and G is the additive genetic covariance matrix, i.e., the (co)variances among individuals of breeding values. The quadratic selection gradient matrix has direct relationships to the change in the distribution of breeding values due to selection, but not with such simple relationships between generations as for the directional selection gradient and the change in the mean (Lande & Arnold 1983). Walsh & Lynch (2018) provide an extended treatment of the various relationships between the summaries of the local fitness function provided by ***β*** and ***γ*** to changes in the distribution of phenotype and breeding values.

Some progress has been made at developing generalised regression model methods for inference of selection gradients. Janzen & Stern (1998) proposed a method for binomial fitness components (e.g., per‐interval survival, mated vs. not mated). The Janzen & Stern (1998) method provides estimates of ***β***, and requires fitting a logistic model with linear terms only, calculating the average derivatives at each phenotypic value observed in a sample, and then standardizing to the relative fitness scale. Morrissey & Sakrejda (2013) expanded Janzen & Stern's (1998) basic approach to arbitrary fitness functions (i.e., not necessarily linear) and arbitrary response variable distributions, retaining the basic idea of numerically averaging the slope (and curvature) of the fitness function over the distribution of observed phenotype. Shaw & Geyer (2010) developed a framework for characterizing the distributions of fitness (and fitness residuals) that arise in complex life cycles, and also showed how the method could be applied to estimate selection gradients by averaging the slope or curvature of the fitness function over the observed values of phenotype in a sample.

Perhaps the simplest fitness function, W(z), that arises in evolutionary theory is a log‐linear model, such that

(4) $$W\left(z\right)=e^{\alpha +b^{\prime }z},$$

where ***α*** is an intercept, and b is a vector of linear regression coefficients on the log scale. The derivative of fitness with respect to phenotype is $\partial W/\partial z^{\prime }=bW\left(z\right)$, and so from equation 1 the selection gradient is

(5) $$\beta =\frac{\int bW(z)p(z)dz}{\int W(z)p(z)dz}=b.$$

Thus, under such a model, the directional selection gradient vector ***β*** is equal to b (see also Lande 1983, Chevin & Hospital 2008, Chevin et al. 2015). The fitness function given by equation 4 is convex (upwardly curved) on the scale of fitness (as opposed to log fitness), and this may be regarded both as a biological feature of such a model, or as a statistical artifact of fitting a model that is linear on the log scale (Schluter 1988, Chevin & Hospital 2008).

More commonly in evolutionary theory (e.g., Lande 1976, Chevin & Haller 2014), Gaussian functions are used, such that

(6) $$W\left(z\right)\propto e^{-\frac{1}{2}\left(z-\theta \right)'\omega^{-1}\left(z-\theta \right)},$$

where θ is the vector of optimal phenotypes and ω describes the curvature; ω must be positive‐definite. Any time that mean trait values are not equal to the optimum (θ), selection in a Gaussian fitness model has a directional component. Specifically,

(7) β=−S(μ−θ),

where μ is mean phenotype and $S={\left(\omega +\Sigma \right)}^{-1}$, where Σ is the phenotypic variance‐covariance matrix before selection; this relation is used extensively in evolutionary theory (e.g., Lande 1976, 1979, Gomulkiesicz & Houle 2009, Chevin & Haller 2014). Quadratic selection gradients are more rarely considered in theory that uses Gaussian fitness functions, perhaps because those functions are seen as general models of stabilizing selection, and therefore represent quite constrained models of nonlinear selection. The quadratic selection gradient matrix is given by

(8) $$\gamma =\beta \beta^{\prime }-S$$

(Chevin et al. 2015; note that this reference gives a mis‐printed version of this relation). The expressions given here for selection gradients in Gaussian fitness models deviate somewhat from typical presentations in so far as we give multivariate expressions for all quantities.

It is possible to fit constrained functions (e.g., Gaussian) to empirical data in studies on natural selection, although this is rarely done (see Chevin et al. 2015 for an excellent example, linking theoretical quantities to selection gradients using a Gaussian model). More general exponential functions may be very useful for empirical studies of natural selection, particularly if they make less restrictive assumptions about the overall form of selection. If the coefficients of more general functions can be related analytically to selection gradients, they may help to better link theoretical and empirical evolutionary quantitative genetic studies. We sought to determine whether analytical relationships could be found between selection gradients and the parameters of exponential functions with quadratic exponents, i.e.,

(9) $$W\left(z\right)=e^{a+\sum_{i}b_{i}z_{i}+\sum_{i}\frac{1}{2}g_{i}z_{i}^{2}+\sum_{i=1}^{k-1}\sum_{j=i+1}^{k}g_{ij}z_{i}z_{j}}.$$

Such functions are easily estimated via generalised linear models, using log link functions, and inherently benefit from the property of exponential functions to treat the response variable (expected fitness in this case) as a strictly positive quantity. The class of function we consider is also a generalization of the Gaussian fitness function (as in eq. 6), and is therefore linked to evolutionary quantitative genetic theory, but provides a more flexible model of nonlinear selection. Specifically, it need not be assumed that the function contains a maximum; when it does, relationships to theory that uses Gaussian functions may be invoked. As such, this more general approach, and its immediate link to statistical analysis with generalized linear models, may be very attractive to empiricists. We obtain analytical links between the regression parameters in equation 9 and ***β*** and ***γ***. Our expressions coincide with known relations for Gaussian fitness functions (i.e., equations 7 and 8). The results are thus a particularly satisfying link between procedures that are likely to be adopted by empiricists, and the kinds of function that are used by theoreticians in evolutionary quantitative genetics. We also provide expressions for biological and statistical variance in selection gradients, given variance in the parameters of the regression in equation 9, and some links between exponential fitness functions and some other analyses about fitness components in use in evolutionary studies.

## Selection Gradients and Exponential Fitness Functions with Quadratic Arguments

Before detailing our results, a brief description of the factor of $\frac{1}{2}$ associated with the quadratic terms in equation 9, analogous to that surrounding a similar factor in Lande & Arnold's (1983) paper (see Stinchcombe et al. 2008), may prevent confusion. In order to obtain the correct values of the $g_{i}$ coefficients, the covariate values for quadratic terms should be squared and then halved. An alternative analysis is possible, where the squared covariate values are not halved, but the estimated coefficient estimates are doubled (analogous to procedures discussed by Stinchcombe et al. 2008). However, this alternative analysis leads to an additional, and potentially confusing, step in the calculation of standard errors and variance‐covariance matrices of selection gradients in replicated studies (detailed in the appendix).

Define a vector $b={\left(b_{1},\text{…},b_{k}\right)}^{\prime }$ containing the coefficients of the linear terms in the exponent of the model in equation 9, and a matrix $g=(g_{ij})$ containing the coefficients of the corresponding quadratic form. We can then write the fitness function more conveniently in matrix form

(10a) $$W\left(z\right)=e^{f(z)}$$

(10b) $$f\left(z\right)=a+b^{\prime }z+\frac{1}{2}z^{\prime }gz.$$

Let **d** be a vector of the expectations of the first order partial derivatives of W(z) and let H be the matrix of expectations of the second order partial derivatives of W(z). Thus the elements of **d** are $d_{i}=E\left[\frac{\partial W(z)}{\partial z_{i}}\right]$ and the elements of H are $H_{ij}=E\left[\frac{\partial^{2}W\left(z\right)}{\partial z_{i}\partial z_{j}}\right]$. We can now rewrite the expressions for directional and quadratic selection gradients as

(11) $$\beta =\frac{d}{E[W(z)]}$$

and

(12) $$\gamma =\frac{H}{E[W(z)]}.$$

Differentiating equation 10 gives

(13) $$\frac{\partial W(z)}{\partial z^{\prime }}=\left(b+gz\right)e^{f(z)},$$

and

(14) $$\frac{\partial^{2}W\left(z\right)}{\partial z\partial z^{\prime }}=\left(g+\left(b+gz\right){\left(b+gz\right)}^{\prime }\right)e^{f(z)}.$$

Assume that the phenotype **z** is multivariate normal, with mean μ and covariance matrix Σ, and denote its probability density by $p_{\mu ,\Sigma }\left(z\right)$. Provided $e^{f(z)}$ has a finite expectation, the function

(15) $$K\left(z\right)=\frac{e^{f(z)}p_{\mu ,\Sigma }\left(z\right)}{E\left[e^{f(z)}\right]}$$

is a probability density function giving the distribution of phenotypes after selection. Define the matrix $\Omega^{-1}=\Sigma^{-1}-g$ and the vector ν=μ+Ω(b+gμ). We show in the Appendix that Ω is symmetric. Provided it is also positive definite, it is a valid covariance matrix, and, by equation A8, $K\left(z\right)\propto p_{\nu ,\Omega }\left(z\right)$. As *K* is a probability density function this implies

(16) $$K\left(z\right)=p_{\nu ,\Omega }\left(z\right).$$

Define $Q^{-1}=\Omega^{-1}\Sigma =I_{k}-g\Sigma .\;$ Combining equations (11), (13) and 16 yields β=E[b+gz], where the expectation is taken with respect to *K*. This is an expectation of a linear function of z, and so

(17) $$\begin{array}{ccc} \beta & = & b+g\nu =\left(b+g\mu \right)+g\Omega \left(b+g\mu \right)=\left(I_{k}+g\Omega \right)\left(b+g\mu \right)\\ & = & Q(b+g\mu ), \end{array}$$

by use of equation A5.

Combining equations (12), (14), and 16 yields $\gamma =E[g+\left(b+gz\right){\left(b+gz\right)}^{\prime }]$, where the expectation is taken with respect to *K*. Hence

(18) $$\begin{array}{c} \begin{array}{ccc} \gamma & = & g+\mathrm{VAR}\left(b+gz\right)+\left[E\left(b+gz\right)\right]{\left[E\left(b+gz\right)\right]}^{\prime }\\ & = & g+g\Omega g^{\prime }+\beta \beta^{\prime }\\ & = & \beta \beta^{\prime }+\left(I_{k}+g\Omega \right)g\\ & = & \beta \beta^{\prime }+Qg, \end{array} \end{array}$$

where we have noted that **g** is symmetric and used equation A5.

In univariate analyses, the matrix machinery necessary for implementing the general formulae in equations 17 and 18 can be avoided. If the fitness function is $W\left(z\right)=e^{a+bz+\frac{1}{2}gz^{2}}$, and *z* has a mean of μ and a variance of σ^2^, then $\beta =\frac{b+g\mu }{1-g\sigma^{2}}$ and $\gamma =\frac{{\left(b+g\mu \right)}^{2}+g\left(1-g\sigma^{2}\right)}{{\left(1-g\sigma^{2}\right)}^{2}}$. These expressions will hold for any univariate analysis, and can be applied to get mean‐standardized, variance‐standardized, and unstandardized selection gradients, when appropriate values of μ and σ^2^ are used, and applied to log‐quadratic models of W(z) where the phenotypic records have been correspondingly standardized. For the common case where the trait is mean‐centred and (unit) variance standardized, the expressions simplify further to $\beta =\frac{b}{1-g}$ and $\gamma =\frac{b^{2}+g\left(1-g\right)}{{\left(1-g\right)}^{2}}$.

The notation we have used is designed to relate the parameters one may estimate directly in a regression analysis to selection gradients. This should facilitate the application of log‐linear models of fitness functions in empirical models of selection. Interestingly, the expressions we obtain, while more general than Gaussian functions (they do not constrain selection to have a stabilising form), coincide with those for Gaussian selection. This equivalence is demonstrated in the appendix. We have thus shown that these expressions are more general and empirically useful than has previously been known. The requirements for obtaining selection gradients from Gaussian and the more general exponential functions with quadratic arguments are slightly different. For Gaussian functions, ω (as in equation 6) must be positive definite. Equations 17 and 18 require that Ω, which depends on both **g** and Σ, is positive definite. In univariate analyses, this condition reduces to $g<\frac{1}{\sigma^{2}}$, implying that the fitness function should not curve upward too sharply within the range of observed phenotype.

The main application of the expressions given here to obtain selection gradient estimates from log‐linear or log‐quadratic arguments is generalized linear model (GLM) regression analyses with log link functions. With appropriate specification of linear, quadratic, and correlational terms, GLMs with any response variable distribution should yield selection gradient estimates, providing that they specify a log link function. This will include, for example, binomial and negative binomial GLM models, with arguments specified as in equation 4 or 9. The equations also apply directly to log‐link models with additive overdispersion (appendix section). Additionally under certain conditions, several other analyses commonly used to assess the dependence of fitness, or fitness components, on quantities such as phenotypic traits can yield log‐linear or log‐quadratic models of trait‐fitness relationships, and can thus be used with the expressions given in this section for ***β*** and ***γ***. These include special cases of parentage analysis, capture‐mark‐recapture analysis, and survival analysis. The conditions under which these methods can yield selection gradient estimates are elaborated in appendix sections.

## Biological Variation and Statistical Uncertainty

The expressions for selection gradients, given the parameters of a log‐quadratic fitness function (equations 17 and 18) give the selection gradients conditional on the estimated values of b and g. However, we often make inference about selection, not merely to quantify selection at a given place and time, but rather to ask larger questions, for example, about how selection varies. For exponential models with a linear argument (equation 4), the variance in b among, for example, cohorts or populations, may be estimated by random‐regression analysis, and would represent the variance in directional selection gradients, since β=b. However, since the *b* and *g* coefficients in an exponential model of fitness with a quadratic argument (equation 9) are not themselves selection gradients, their variances and covariances are not the variances and covariances of ***β*** and ***γ***. Moreover in empirical studies of natural selection, b and g will not typically be known quantities, but rather will be estimates with error.

Whether variances and covariances of the elements of **b** and **g** are of biological interest (e.g., variation among temporal replicates of a selection analysis), or are statistical (e.g., sampling variance), the corresponding biological or sampling variances of ***β*** and ***γ*** can potentially be obtained by integrating approximations, bootstrapping, and/or Monte Carlo methods. In particular, approximation of variances in ***β*** and ***γ*** by a first‐order Taylor approximation (the “delta method”; Lynch & Walsh 1998) may generally be pragmatic. Formulae for approximate biological or statistical variances of ***β*** and ***γ*** given covariances of **b** and **g** by this method are given in the appendix. For univariate analysis, with phenotype standardised to μ=0 and $\sigma^{2}=1$, the approximate variances β and γ are given by

(19) $$\mathrm{Var}\left[\beta \right]\approx \frac{Var[b]}{{\left(1-g\right)}^{2}}+\frac{b^{2}Var\left[g\right]}{{\left(1-g\right)}^{4}}+\frac{2bCov[b,g]}{{\left(1-g\right)}^{3}},$$

and

(20) $$\mathrm{Var}\left[\gamma \right]\approx \frac{4b^{2}Var\left[b\right]}{{\left(1-g\right)}^{4}}+\frac{{\left(1+2b^{2}-g\right)}^{2}Var\left[g\right]}{{\left(1-g\right)}^{6}}+\frac{4b\left(1+2b^{2}-g\right)Cov\left[b,g\right]}{{\left(1-g\right)}^{5}},$$

where Var[b] and Var[g] represent the variances of *b* and *g* terms and Cov[b,g] is the covariance of the *b* and *g* terms. These variances and covariances may represent real variation in trait‐fitness relationships (e.g., variation in time or space), or they may represent statistical uncertainty in parameter values. If equations 19 and 20 are used to represent statistical uncertainty in estimates $\hat{\beta }$ and $\hat{\gamma }$, Var[b] would be replaced by $Var[\hat{b}]$ (the sampling variance, or squared standard error of the estimate of *b*), etc. The sampling covariance of *b* and *g* (i.e., $Cov[\hat{b},\hat{g}]$) is not routinely reported by most statistical software packages, but can generally be obtained (the use of the sampling covariance of $\hat{b}$ and $\hat{g}$ terms in a GLM model in R is demonstrated in the supplemental materials).

### STATISTICAL UNCERTAINTY: SIMULATION

We performed a small simulation study to assess the extent of any bias in the estimators β and γ and the adequacy of the first‐order approximation of their standard errors. We simulated univariate directional selection, with values of *b* between ‐0.5 and 0.5, and with g=−1,0, and 0.4. Because β and γ are nonlinear functions of *g*, it is not possible to simultaneously investigate ranges of parameter values with regular intervals of values of both *g* and selection gradients. These values of *g* represent a compromise between investigating a regular range of *g* and γ. We used a (log) intercept of the fitness function of a=0. We simulated a sample size of 200 individuals. This sample size reflects a very modest‐sized study with respect to precision in inference of nonlinear selection, and is therefore a useful scenario in which to judge performance of different methods for calculating standard errors. Fitness was simulated as a Poisson variable with expectations defined by the ranges of values of *b* and *g*, and with phenotypes sampled from a standard normal distribution.

First, we analyzed each simulated dataset using the OLS regression described by Lande & Arnold (1983), i.e., $w_{i}=\mu +\beta z_{i}+\frac{1}{2}\gamma z_{i}^{2}+e_{i}$, using the R function lm(). For the OLS regressions, we calculated standard errors assuming normality using the standard method implemented in the R function summary.lm(), and by case‐bootstrapping, by generating 1000 bootstrapped datasets by sampling with replacement, running the OLS regression analysis, and calculating the standard deviation of the bootstrapped selection gradient estimates. Second, we fitted a Poisson GLM with linear and quadratic terms, using the R function glm(). We then calculated conditional selection gradient estimates using equations 17 and 18. We obtained standard errors by using a first‐order Taylor series approximation (the “delta method”; Lynch & Walsh 1998, appendix A1). For each method of obtaining estimates and standard errors, we calculated the standard deviation of replicate simulated estimates. We could thus evaluate the performance of different methods of obtaining standard errors by their ability to reflect this sampling standard deviation. We also calculated mean absolute errors for both estimators of β and γ for all scenarios. Every simulation scenario and associated analysis of selection gradients was repeated 1000 times.

**Figure 1:**
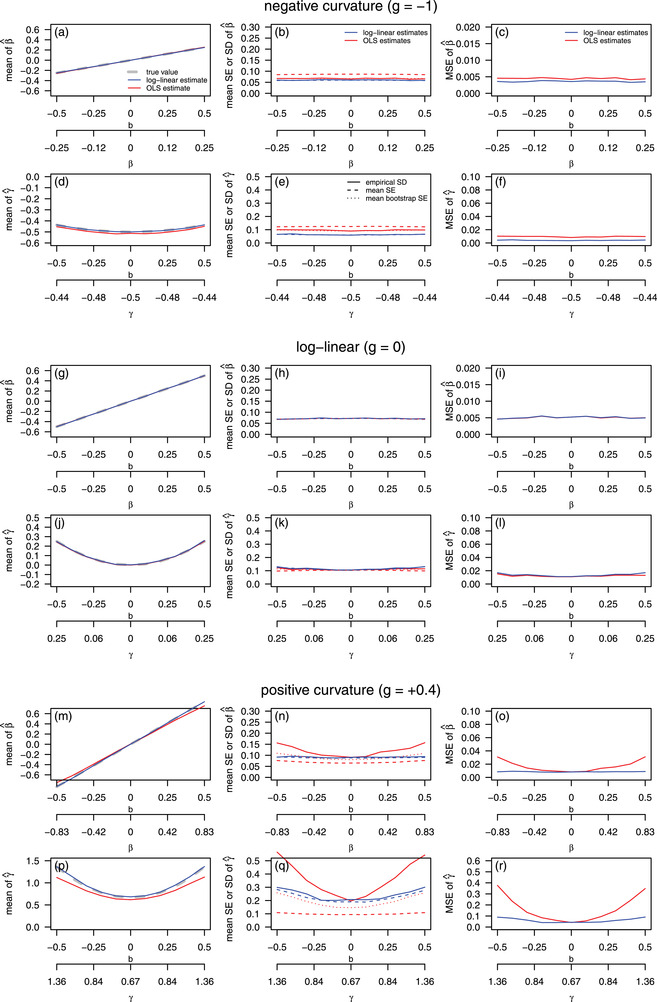
Simulation results for the performance of Lande & Arnold's (1983) least squares‐based (OLS) estimators (red lines), and log‐quadratic (GLM) estimators (blue lines), of directional and quadratic selection gradients. The first column shows bias in estimates of β and γ, where departure from the grey line (the simulated truth) indicates bias. The middle column shows the performance of OLS standard errors (red dashed lines), bootstrap standard errors (red dotted lines), and first‐order approximations (blue dashed lines) of the standard errors of the GLM estimators. Ideally, all values of estimated mean standard errors would fall on the simulated standard deviation of their associated estimators, shown as solid lines. The right column shows the mean squared errors of the OLS and GLM estimators. Note that the *y*‐axis scale for the MSE in plot o differs from that in plots (c) and (i), that the scale in plot (r) differs from that in plots (f) and (l), and that the scales in plots (d), (j), and (p) differ.

Selection gradient estimates obtained by all three methods were essentially unbiased (Figure 1a,d,g,j,m,p), except for small biases that occurred when the fitness function was very curved. Thus, GLM‐derived values of selection gradients, conditional on estimated values of *b* and *g*, performed very well as estimators of β and γ in our simulations. Similarly, first‐order approximations of standard errors of the GLM‐derived estimates of β and γ closely reflected the simulated standard deviations of the estimators (Figure 1b,e,h,k,n,q). All methods for obtaining standard errors performed well for estimates of β in the pure log‐linear selection simulations (Figure 1h,k). OLS standard errors performed reasonably well under most simulation scenarios, except when *g* was positive (Figure 1n,q); across all scenarios bootstrap standard errors of the OLS estimators outperformed OLS standard errors produced using the standard formula. Mean squared error of the GLM estimators was always smaller than that of the OLS estimators of β and γ. This is unsurprising, as the simulation scheme corresponded closely to the GLM model. These results demonstrate the usefulness of the conditional values of β and γ as estimators, and show that gains in precision and accuracy can be obtained when glm models of fitness functions fit the data well.

It remains plausible that the OLS estimators motivated by Lande & Arnold's (1983) work could outperform glm‐based analyses in some scenarios. In particular, the OLS estimators will rarely be misleading (Figure 1), whereas the risk of bias or misleading standard errors in workflows involving GLMs resulting from, for example, mis‐specification of error structures, is not known. What the theory and limited simulations presented here demonstrate is that (a) given a well‐specified log‐link GLM, selection gradients can be obtained and (b) first‐order approximations to the standard errors of these GLM‐based selection gradients seem to perform adequately.

### VARIATION IN SELECTION: EMPIRICAL EXAMPLE

In order to explore the behavior of estimates of ***β*** and ***γ*** derived from log‐quadratic fitness models, we conducted a small study of selection and variation in selection of birth mass in female Soay sheep (*Ovis aries*), from a long‐term study on St Kilda, in the Outer Hebrides, Scotland (Clutton‐Brock & Pemberton 2004). Briefly, the data comprise records of mass taken within 5 days of birth, and subsequent lifetime breeding success (i.e., the total number of offspring produced, regardless of the fates of those offspring) of females from cohorts born between 1985 and 2006 (inclusive, except for 2001, when data collection was suspended due to precautions associated with an outbreak of foot‐and‐mouth disease). Fitness data were collected between 1985 and 2016. A small number of ewes from later cohorts (i.e., 2005 and 2006) will have still been alive in 2016, and their fitness will therefore be slightly underestimated. Birth masses were corrected for growth in the first 5 days prior to other analyses by fitting a linear model of mass as a function of age, and then correcting mass for growth.

We fitted a single model as our basis for estimating the variance in selection among cohorts. We fitted a generalized linear random regression mixed model describing the dependence of lifetime breeding success, $W_{ij}$, of individual *i* from cohort *j*, as a function of their birth mass $z_{ij}$, according to

(21a) $$\begin{array}{cc} W_{ij} & =P\left(E{\left[W\right]}_{ij}\right), \end{array}$$

(21b) $$\begin{array}{cc} log\left(E{\left[W\right]}_{ij}\right) & =a_{j}+\bar{b}z_{ij}+\frac{1}{2}\bar{g}z_{ij}^{2}+\delta_{b,j}z_{ij}^{2}+\frac{1}{2}\delta_{g,j}z_{ij}+e_{ij}, \end{array}$$

where P(λ) denotes samples from a Poisson distribution with expectation λ, $a_{j}$ are cohort‐specific intercepts, and $\bar{b}$ and $\bar{g}$ are overall log‐scale slopes and curvatures. $\delta_{b,j}$ and $\delta_{g,j}$ are cohort‐specific random effects for slopes and curvature terms, assumed to be distributed according to

$${\left[\begin{array}{c} \delta_{b}\\ \delta_{g} \end{array}\right]}_{j}∼N\left(0,\Sigma_{\delta }\right),$$

and elements of the covariance matrix $\Sigma_{\delta }=\left[\begin{array}{cc} \sigma_{\delta_{b}}^{2} & \sigma_{\delta_{b},\delta_{g}}\\ \sigma_{\delta_{b},\delta_{g}} & \sigma_{\delta_{g}}^{2} \end{array}\right]$ are estimated. $e_{ij}$ are residuals, with zero means and cohort‐specific variances. We implemented the model defined by equations 21 as a mixed model, and collected MCMC samples, using MCMCglmm (Hadfield 2010). We used diffuse normal priors on the fixed effects, an inverse‐Wishart prior for $\Sigma_{\delta }$, and inverse‐gamma priors on the cohort‐specific log‐scale overdispersion variances, $\sigma_{e}^{2}$. We used informative priors on the cohort‐specific overdispersion variances, $\sigma_{e}^{2}$, in order to help the model to predict cohort‐specific mean fitnesses that agreed well with the observed data. The specific model is fully detailed in the code supplement.

**Figure 2:**
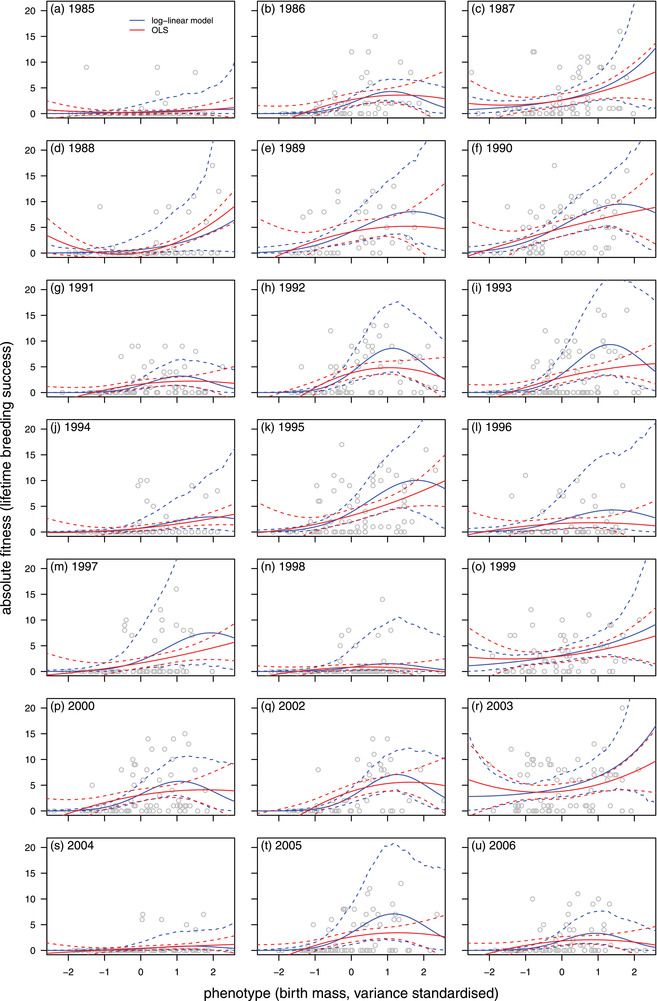
Estimates of functions relating birth mass to lifetime breeding success in 21 cohorts of Soay sheep (*Ovis aries*) on St Kilda from 1985 to 2006; note that inference of selection in 2001 is not possible because very little phenotypic data were collected on account of the foot and mouth outbreak in the UK that year. Estimated fitness functions are given with 95% confidence intervals (OLS) or credible intervals (Bayesian generalized linear mixed model or GLMM) intervals of the predictions. OLS estimates are generated independently for each cohort; GLMM estimates depict cohort‐specific predictions from the random regression mixed model described in equation 21.

Selection is predominantly directional, favoring larger birth masses (Table 1). Quadratic selection is near zero on average, but not because there is generally a lack of curvature of the fitness function. The combination of positive values of *b* and negative values of *g* (from fitting equation 21; Table 1 a) implies that the fitness function is often upwardly curved over much of the distribution of birth mass, but becomes downwardly curved in the region of large birth masses (Figure 2), such that the curvature, averaged over the distribution of phenotype, tends to be near zero. Using the system given in the appendix for calculating variances in β and γ (with univariate cases given in equations 19 and 20), we inferred that there is very substantial variation in both directional and quadratic selection. While β varies greatly (${\hat{\sigma }}_{\beta }$ = 0.18 (0.04 –  0.34); Table 1 b), because selection is so strongly directional, this does not lead to fluctuations in the direction of selection. The fact that the average quadratic selection gradient is near zero does not imply that nondirectional selection is absent. Rather, in contrast to the situation for directional selection, the estimate of ${\hat{\sigma }}_{\gamma }=0.35\;\left(0.06-\;0.71\right)$ (Table 1 b), given that ${\hat{\mu }}_{\gamma }$ = 0.02 (–0.21 –  0.21), suggests quadratic selection varies substantially, sometimes taking positive values, and sometimes being negative.

**Table 1:** Parameters of the random regression mixed model (a) used to characterize variation in selection of lamb body mass in Soay sheep (*Ovis aries*). Part (b) gives estimates of the means, variances, and covariances of selection gradients, derived from the model reported in part (a). For ease of interpretation, estimates of variation in β and γ are reported as standard deviations, and the relationship between β and γ is reported as a correlation

Parameter	Estimate and 95% CI
(a) Mixed model parameters	
*b*	1.07 ( 0.83 – 1.35)
*g*	−0.72 (−1.08 – ‐0.33)
$\sigma_{d}^{2}$	0.25 (0.05 – 0.50)
$\sigma_{e}^{2}$	0.47 (0.07 – 1.02)
$\sigma_{d,e}$	−0.26 (−0.56 – 0.00)
(b) Derived parameters of the distribution of selection gradients	
$\mu_{\beta }$	0.62 ( 0.52 – 0.74)
$\mu_{\gamma }$	0.02 (‐0.21 – 0.21)
$\sigma_{\beta }$	0.18 ( 0.04 – 0.34)
$\sigma_{\gamma }$	0.35 ( 0.06 – 0.71)
$\rho_{\beta ,\gamma }$	0.64 (‐0.18 – 1.00)

Annual (log) quadratic functions from the random regression mixed model (equation 21) correspond reasonably closely to quadratic OLS estimates, for most cohorts (Figure 2). There are some systematic differences, arising primarily from the fact that the two models have different functional forms; the former is a quadratic function, while the latter is a quadratic function on the log scale, or a Gaussian function when the parameter *g* is negative, as it often is (Table 1 a).

## Conclusion

We have provided analytical expressions for selection gradients, given the parameters of exponential fitness models with quadratic arguments. These functions can be applied in conjunction with a range of generalised linear model approaches, specific situations in capture‐mark‐recapture, survival analysis, and parentage analysis, and relate empirical selection gradients directly to types of fitness functions used in theoretical studies. The general relationship of selection gradients to the coefficients of log‐linear and log‐quadratic models, and in particular, various ways of estimating these using generalized linear models, are probably the most generally useful feature of our results. In empirical applications, our preliminary simulation results indicate that, given an appropriate model of a log‐scale fitness function, inference using log‐linear and log‐quadratic models may be very robust, and could provide more reliable statements about uncertainty (i.e., reasonable standard errors) than the main methods used to date. It should be noted, however, that OLS methods proved to be highly robust in our simulations with Poisson fitness residuals (see also McGee, submitted), except in the presence of very strong nonlinear selection. Furthermore, the relationships given here between log‐quadratic fitness functions and selection gradients could lead to better integration between empirical and theoretical strategies for modelling selection.

## AUTHOR CONTRIBUTIONS

MBM and IBJG developed the theory, conducted the analyses, and wrote the manuscript together.

Associate Editor: J. Tufto

Handling Editor: A. G. McAdam

## Supporting information

Supporting Information

## GAUSSIAN FITNESS FUNCTIONS AS SPECIAL CASES OF LOG NORMAL FITNESS FUNCTIONS WITH QUADRATIC ARGUMENTS

Suppose that the fitness function W(z) is proportional to a Gaussian density function for z with mean ***θ*** and covariance matrix ***ω***. The exponent of the corresponding exponential function is then given by

$$-\frac{1}{2}{\left(z-\theta \right)}^{\prime }\omega^{-1}\left(z-\theta \right)=-\frac{1}{2}\theta^{\prime }\omega^{-1}\theta +\theta^{\prime }\omega^{-1}z-\frac{1}{2}z^{\prime }\omega^{-1}z=a+b^{\prime }z+\frac{1}{2}z^{\prime }gz,$$

where $a=-\frac{1}{2}\theta^{\prime }\omega^{-1}\theta$, $b^{\prime }=\theta^{\prime }\omega^{-1}$ and $g=-\omega^{-1}$. Let z have a Gaussian distribution with mean ***μ*** and covariance matrix Σ. Setting $S={\left(\omega +\Sigma \right)}^{-1}$, the selection gradient vector ***β*** given by equation 7 is

(A1)
$$\begin{array}{cc} -S\left(\mu -\theta \right)={\left(\omega +\Sigma \right)}^{-1}\left(\omega b-\mu \right) & ={\left(-g^{-1}+\Sigma \right)}^{-1}\left(-g^{-1}b-\mu \right)\\ & ={\left(g^{-1}-\Sigma \right)}^{-1}g^{-1}g\left(g^{-1}b+\mu \right)\\ & ={\left(I_{k}-g\Sigma \right)}^{-1}\left(b+g\mu \right)\\ & =Q(b+g\mu ), \end{array}$$

where Q is defined following equation 16. This shows that the result for the selection gradient using Gaussian fitness functions is a special case of our result in equation 17.

Comparison of the pre‐multipliers of ***μ*** in A1 indicates that −S=Qg. It follows that equation 8, giving the result for quadratic selection gradients using Gaussian fitness functions, is a special case of our result in equation 18.

## SAMPLING VARIANCES OF SELECTION GRADIENT ESTIMATES

Denote a vector containing all unique elements of ***γ*** by $\tilde{\gamma }$. The following assumes that $\tilde{\gamma }$ is composed by vertically stacking the columns of the diagonal and sub‐diagonal elements of ***γ***. For example, in an analysis with three traits, $\tilde{\gamma }={\left[\begin{array}{c} \gamma_{1,1},\gamma_{2,1},\gamma_{3,1},\gamma_{2,2},\gamma_{3,2},\gamma_{3,3} \end{array}\right]}^{\prime }$. Let v() denote the function mapping the distinct elements of a symmetric matrix r onto the column vector $\tilde{r}$.

The first‐order approximation to the sampling covariance matrix of the elements of ***β*** and ***γ*** is then given by $J\tilde{\Sigma }J^{\prime }$, where $\tilde{\Sigma }$ is the sampling covariance matrix of a vector containing the elements of b and $\tilde{g}$, where the latter is a column vector containing the distinct elements of g arranged according to the same scheme that defines $\tilde{\gamma }$. J is the Jacobian, or gradient matrix of first order partial derivatives, of ***β*** and $\tilde{\gamma }$ with respect to b and $\tilde{g}$, i.e.,

$$J=\left[\begin{array}{cc} \frac{\partial \beta }{\partial b} & \frac{\partial \beta }{\partial \tilde{g}}\\ \frac{\partial \tilde{\gamma }}{\partial b} & \frac{\partial \tilde{\gamma }}{\partial \tilde{g}} \end{array}\right],$$

evaluated at the estimated values of b and g.

Note that some users may prefer to fit the model 9 with $g_{ii}$ replaced by $2g_{i}$, say. The formulae for β and γ are readily re‐expressed in terms of these variables by making this substitution. If $\Sigma_{1}$ denotes the covariance matrix obtained when fitting this revised model, the required covariance matrix $\tilde{\Sigma }$ can be calculated using $\tilde{\Sigma }=D\Sigma_{1}D^{\prime }$, where **D** is a diagonal matrix with all the diagonal elements equal to one, apart from those corresponding to the variables $g_{ii}$ which equal 2.

The four submatrices of J can be treated separately. Noting that β=Q(b+gμ) (equation 17),

(A2)
$$\frac{\partial \beta }{\partial b}=Q.$$

Let $s=\frac{1}{2}k\left(k+1\right)$, where *k* is the number of traits in the analysis, and let $e_{1},\cdots ,e_{s}$ be the standard basis for an *s* dimensional space (i.e., $e_{1}={\left[1,0,\cdots ,0\right]}^{\prime }$, etc.). Define an indicator matrix $C_{m}=C^{\left(i,j\right)}$ where $C^{\left(i,j\right)}$ is a *k* by *k* matrix in which

$${\left[C^{\left(i,j\right)}\right]}_{xy}=\left\{\begin{array}{cc} 1, & (x,y)=(i,j)\;\text{or}\;(j,i);\\ 0, & \text{otherwise}. \end{array}\right.$$

Using the standard expression for the derivative of the inverse of a matrix with respect to a scalar, we can obtain $\frac{\partial \beta }{\partial \tilde{g}}$, i.e., the upper‐right sub‐matrix of J.

(A3)
$$\begin{array}{ccc} \beta =\Psi^{-1}\left(b+g\mu \right)\;\Rightarrow \frac{\partial \beta }{\partial {\tilde{g}}_{m}} & = & \frac{\partial \beta }{\partial g_{ij}}=-\Psi^{-1}\left[\frac{\partial \Psi }{\partial g_{ij}}\right]\Psi^{-1}\left(b+g\mu \right)+\Psi^{-1}\left[\frac{\partial \left(b+g\mu \right)}{\partial g_{ij}}\right]\\ & = & -Q\left[\frac{\partial \;I_{k}-g\Sigma }{\partial g_{ij}}\right]Q\left(b+g\mu \right)+Q\left[\frac{\partial \;g}{\partial g_{ij}}\right]\mu \\ & = & Q\left[\frac{\partial g}{\partial g_{ij}}\right]\left[\Sigma Q\left(b+g\mu \right)\right]+Q\left[\frac{\partial \;g}{\partial g_{ij}}\right]\mu \\ & = & QC^{\left(ij\right)}\left(\Sigma \beta +\mu \right)=QC_{m}\left(\Sigma \beta +\mu \right)\\ \Rightarrow \frac{\partial \beta }{\partial \tilde{g}} & = & \sum_{m=1}^{s}\frac{\partial \beta }{\partial {\tilde{g}}_{m}}e_{m}^{\prime }=Q\sum_{m=1}^{s}C_{m}\left(\Sigma \beta +\mu \right)e_{m}^{\prime } \end{array}$$

Let $Q_{\left[u\right]}$ denote the *u* ^th^ column of **Q**. Using the previous relation $\frac{\partial \beta }{\partial b}=Q$, we can obtain $\frac{\partial \tilde{\gamma }}{\partial b}$, i.e., the lower‐left sub‐matrix of J.

(A4)
$$\begin{array}{cccc} \gamma ={\beta \beta }^{\prime }+\mathrm{Qg} & & \Rightarrow \frac{\partial \gamma }{\partial b_{u}} & =\beta {\left(\frac{\partial \beta }{\partial b_{u}}\right)}^{\prime }+\left(\frac{\partial \beta }{\partial b_{u}}\right)\beta^{\prime }=\beta Q_{\left[u\right]}^{\prime }+Q_{\left[u\right]}\beta^{\prime }\\ & & \Rightarrow \frac{\partial \tilde{\gamma }}{\partial b_{u}} & =v\left(\beta Q_{\left[u\right]}^{\prime }+Q_{\left[u\right]}\beta^{\prime }\right)\\ & & \Rightarrow \frac{\partial \tilde{\gamma }}{\partial b} & =\sum_{u=1}^{k}v\left(\beta Q_{\left[u\right]}^{\prime }+Q_{\left[u\right]}\beta^{\prime }\right)e_{u}^{\prime } \end{array}$$

Let $M^{\left(m\right)}=QC_{m}\left(\Sigma \beta +\mu \right)\beta^{\prime }$. Note that $Q^{-1}=\Omega^{-1}\Sigma$ implies Ω=ΣQ. Moreover $\Omega^{-1}=\Sigma^{-1}-g$ implies firstly that

(A5)
$$I_{k}+g\Omega =\Sigma^{-1}\Omega =Q$$

and secondly that Ω is symmetric, since Σ and g are both symmetric. It follows that

(A6)
$$Q^{\prime }=I_{k}+{\left(g\Omega \right)}^{\prime }=I_{k}+\Omega g.$$

The lower‐right sub‐matrix of J can then be derived.

(A7)
$$\begin{array}{c} \begin{array}{cc} \frac{\partial \gamma }{\partial g_{ij}} & =\left[\frac{\partial \beta }{\partial g_{ij}}\right]\beta^{\prime }+\beta {\left[\frac{\partial \beta }{\partial g_{ij}}\right]}^{\prime }+{\mathrm{QC}}^{\left(ij\right)}+{\mathrm{QC}}^{\left(ij\right)}\Sigma \mathrm{Qg}\\ & =\left[QC^{\left(ij\right)}\left(\Sigma \beta +\mu \right)\right]\beta^{\prime }+\beta {\left[QC^{\left(ij\right)}\left(\Sigma \beta +\mu \right)\right]}^{\prime }+{\mathrm{QC}}^{\left(ij\right)}+{\mathrm{QC}}^{\left(ij\right)}\Omega g\\ \Rightarrow \frac{\partial \tilde{\gamma }}{\partial g_{ij}} & =v\left[M^{\left(m\right)}+{\left(M^{\left(m\right)}\right)}^{\prime }+{\mathrm{QC}}_{m}\left(I_{k}+\Omega g\right)\right]\\ \Rightarrow \frac{\partial \tilde{\gamma }}{\partial \tilde{g}} & =\sum_{m=1}^{s}v\left[M^{\left(m\right)}+{\left(M^{\left(m\right)}\right)}^{\prime }+{\mathrm{QC}}_{m}Q^{\prime }\right]e_{m}^{\prime }, \end{array} \end{array}$$

by use of equation A6.

Finally note that equations A5 and A6 are also relevant to the derivation of formula 16. By definition, $f\left(z\right)=a+z^{\prime }b+\frac{1}{2}z^{\prime }gz$, and we have $\log\left[p_{\mu ,\Sigma }\left(z\right)\right]=-\frac{1}{2}z^{\prime }\Sigma^{-1}z+z^{\prime }\Sigma^{-1}\mu +\alpha$, where α does not depend on **z**. Thus, if $\alpha^{\prime }=\alpha +a$, it follows that, as a function of **z**,

$$\begin{array}{ccc} f\left(z\right)+\log[p_{\mu ,\Sigma }\left(z\right)] & = & -\frac{1}{2}z^{\prime }\left(\Sigma^{-1}-g\right)z+z^{\prime }\left(b+\Sigma^{-1}\mu \right)+\alpha^{\prime }\\ & = & -\frac{1}{2}z^{\prime }\Omega^{-1}z+z^{\prime }\Omega^{-1}\left[\Omega (b+\Sigma^{-1}\mu )\right]+\alpha^{\prime }, \end{array}$$

Now, by A5 and A6, we have $\Omega \left(b+\Sigma^{-1}\mu \right)=\Omega b+{\left(\Sigma^{-1}\Omega \right)}^{\prime }\mu =\Omega b+Q^{\prime }\mu =\Omega b+\left(I_{k}+\Omega g\right)\mu =\nu$, implying that

(A8)
$$\begin{array}{ccc} f\left(z\right)+\log[p_{\mu ,\Sigma }\left(z\right)] & = & -\frac{1}{2}z^{\prime }\Omega^{-1}z+z^{\prime }\Omega^{-1}\nu +\alpha^{\prime }\\ & = & -\frac{1}{2}{\left(z-\nu \right)}^{\prime }\Omega^{-1}\left(z-\nu \right)+\alpha^{\prime \prime }, \end{array}$$

where $\alpha^{\prime \prime }$ is constant as a function of **z**. The exponent of $e^{f(z)}p_{\mu ,\Sigma }\left(z\right)$ is thus identical, as a function of **z**, to that of $p_{\nu ,\Omega }\left(z\right)$. Hence formula 16 holds.

## OTHER ANALYSES THAT CORRESPOND TO LOG‐LINEAR AND LOG‐QUADRATIC FITNESS FUNCTIONS

In addition to generalised linear models with log link functions, there may be other cases where models of trait‐fitness relationships may correspond to log‐linear or log‐quadratic fitness functions. In this section, we describe how the formulae for ***β*** and ***γ*** given in equations 17 and 18 are applicable in four kinds of analyses. In each of these situations, it may not be immediately apparent that a form of the fitness function equivalent to equation 9 is implied.

### Models with overdispersion

Key applications of equations to calculate selection gradients from parameters of log‐linear fitness models, such as GLM and generalised linear mixed model (GLMM) analysis, are likely to involve fitness measures with more dispersion than is accommodated by standard statistical distributions such as the Poisson distribution. Multiplicative overdispersion of count variables is often handled with a negative binomial distribution, parameterised via its expectation and a dispersion parameter. Thus negative binomial GLM analyses can be used directly to estimate b and g, and thus ***β*** and ***γ***. For additive overdispersion, the relations between a generalised model of a fitness function and selection gradients are equally direct, but this is not so immediately obvious. Consider a model for expected fitness such as

$$log\left(E{\left[W\right]}_{i}\right)=a+bz_{i}+\frac{1}{2}gz_{i}^{2}+\varepsilon_{i},\;\;\varepsilon_{i}∼N\left(0,\sigma_{\varepsilon }^{2}\right)\;,$$

which represents a GLMM analysis with additive overdispersion. Heterogeneity in the $\varepsilon_{i}$ affects the expectation, such that $E{\left[W\right]}_{i}=e^{a+bz_{i}+\frac{1}{2}gz_{i}^{2}+\frac{1}{2}\sigma_{\varepsilon }^{2}}$ (note that the mean of a log normal distribution depends on the dispersion on the log scale such that $\mu_{x}=e^{\mu_{log_{x}}+\frac{\sigma_{log(x)}^{2}}{2}}$; Aitchison & Brown 1957). In this situation, the selection gradients, β and γ are again given by equations 17 and 18, even though this expression for log fitness or expected fitness ostensibly differs from that implied by equations 10 a,b. This can be seen by rearranging such that the dependence of expected absolute fitness on the dispersion parameter, $\sigma_{\varepsilon }^{2}$, is absorbed into the intercept,

$$E{\left[W\right]}_{i}=e^{\alpha +bz_{i}+\frac{1}{2}gz_{i}^{2}}\;,$$

where

$$\alpha =a+\frac{1}{2}\sigma_{\varepsilon }^{2}\;.$$

Since selection gradients do not depend on the intercept *a* in equations 17 and 18, they do not depend on α in the above expression, and therefore depend neither on the intercept *a* nor the dispersion parameter $\sigma_{\varepsilon }^{2}$. Consequently, linear and quadratic terms from log‐link GLMMs with additive overdispersion can also be used with equations 17 and 18 to obtain selection gradients for Gaussian traits.

### Multinomial models, as in parentage analysis

In parentage inference, some methods have been proposed wherein the probability that candidate parent *i* is the parent of a given offspring is modelled according to

$$W\left(z_{i}\right)\propto e^{f(z_{i})},$$

and where realised parentages of a given offspring array are then modelled according to a multinomial distribution, potentially integrating over uncertainty in paternity assignments based on molecular data (Hadfield et al. 2006, Smouse et al. 1999). When f(z) is a linear function, (Smouse, Meagher & Korbak 1999; T. Meagher, personal communication) interpreted the analysis as being analogous to that of Lande & Arnold (1983), but not necessarily identical. For a linear f(z), this analysis does in fact yield estimates of ***β***, and for a quadratic function, directional and quadratic selection gradients can be obtained using equations 17 and 18. This can be seen by noting that expected fitness, given phenotype, of candidate fathers for any given offspring array will be, in the log‐linear case,

$$W\left(z\right)=ce^{a+b^{\prime }z}\propto e^{\tilde{a}+b^{\prime }z},$$

where *c* is a constant and $\tilde{a}=a+log\left(c\right)$. As W(z) has been expressed in the form of equation 4, it follows from equation 5 that β=b. Moreover, if expected parentage is modelled as a log‐quadratic function, equations 17 and 18 can be used to recover selection gradients from multinomial parentage models.

### Mark‐recapture with constant survival functions

Another case where our formulae may be applicable pertains to inferences of survival rate. Often, data about trait‐dependent survival rates may be assessed over discrete intervals. While the experimental unit of time may be an interval (e.g., a day or a year), the biologically‐relevant aspect of variation in survival may be longevity, i.e., for how many intervals an individual survives. One such situation arises when per‐interval survival rate is assessed via a logistic regression analysis, and trait‐dependent survival rates are (or may be assumed to be) constant across intervals. A common case of logistic regression analysis that satisfies this first condition is often implemented in capture‐mark‐recapture procedures. Suppose that per‐interval survival rate, given phenotype, may be assumed to be constant, with death in a particular interval of an individual with phenotype z occurring at probability ρ(z). If fitness may reasonably be assumed to be reflected by the expected number of intervals survived, then it will be given, as the expectation of a geometric distribution, by

$$W\left(z\right)=\frac{1-\rho (z)}{\rho (z)}.$$

If trait‐dependent per‐interval survival probability is denoted ϕ(z) (ϕ being the standard symbol for survival rate in capture‐mark‐recapture analyses; Lebreton et al. 1992), then the fitness function in terms of expected number of intervals lived is $W\left(z\right)=\frac{1-(1-\phi (z))}{1-\phi (z)}=\frac{\phi (z)}{1-\phi (z)}$. If per‐interval survival rate has been modeled as a logistic regression, i.e.,

$$\phi \left(z\right)=\frac{e^{f(z)}}{1+e^{f(z)}}\;,$$

where ϕ(z) denotes the per‐interval fitness function, and f(z) is the fitness function on the logistic scale, then the fitness function on the discrete longevity scale is

$$W\left(z\right)=\frac{\frac{e^{f(z)}}{1+e^{f(z)}}}{1-\frac{e^{f(z)}}{1+e^{f(z)}}}=e^{f(z)}.$$

Therefore, if f(z) is a linear function, then its terms are the directional selection gradients on the discrete‐longevity scale. If f(z) is a quadratic function, then the corresponding directional and quadratic selection gradients, again if the relevant aspect of fitness is the number of intervals survived, can be obtained using equations 17 and 18. Waller and Svensson (2016) take advantage of these relationships to compare inference of trait‐dependent survival in capture‐mark‐recapture models to classical inference using Lande & Arnold's (1983) least‐squares regression analysis where fitness is assessed as the number of intervals that individuals survive.

It must be stressed that these results do not justify interpretation of logistic regression coefficients of survival probability as selection gradients in a general way. Such coefficients differ from selection gradients for three reasons: (1) they pertain to a linear predictor scale, and natural selection plays out on the data scale, (2) they directly model absolute fitness, not relative fitness, and (3) they pertain to per‐interval survival, which may not necessarily be the aspect of survival that best reflects fitness in any given study. It is only when the number of intervals survived is of interest (and phenotype‐dependent survival rates can be assumed to be constant across intervals) that these three different aspects of scale cancel out such that the parameters of a logistic regression are selection gradients.

### Survival analysis

Another situation where an important analysis for understanding trait‐fitness relationships that has an immediate—but not necessarily immediately apparent—relationship to selection gradients, arises in survival analysis. In a proportional hazards model (Cox 1972), the instantaneous probability of mortality experienced by live individuals, the hazard λ(t), as a function of their phenotype, could be modelled as

$$\lambda \left(t\right)=\lambda_{0}e^{f(z)}\;,$$

where λ_0_ is the baseline hazard, and the factor $e^{f(z)}$ describes individual deviations from this baseline hazard. If the baseline hazard is constant in time, then survival distributions conditional on phenotype are exponential, and have mean $\lambda^{-1}$. So, if fitness may be assumed to be proportional to longevity (as a continuous variable now, not discrete number of intervals as in the relations given above between logistic models of per‐interval survival and selection gradients) then

$$W\left(z\right)\propto \frac{1}{\lambda_{0}e^{f(z)}}=\frac{1}{\lambda_{0}}e^{-f(z)}.$$

In expressions for selection gradients (equations 1 and 2), $\frac{1}{\lambda_{0}}$ would be a constant in the integrals in both the numerators and denominators, and therefore cancels in calculations of selection gradients. Therefore, if proportional hazards are modeled with f(z) as a linear or quadratic function, then the expressions for selection gradients (equations (5), (17) and 18) hold, but the coefficients of the trait‐dependent hazard function must be multiplied by ‐1. The relationship to survival analysis will also approximately hold when fitness is determined by survival to a specific age, and mean survival to that age is low.
