# Supplementary material for: Analytical results for directional and quadratic selection gradients for log‐linear models of fitness functions
Source: Evolution. 2022 May 18;76(7):1378–90. doi: 10.1111/evo.14486 (PMC9546161; doi:10.1111/evo.14486)
Supplement: Supplementary file 1 — Supporting Information [file EVO-76-1378-s001.pdf]

Supplementary Information for: Analytical results for directional and quadratic selection gradients for log-linear models of fitness functions

Sam McGee and Robert W. Service

09/04/2021

General functions to calculate selection gradients and their standard errors

Packages needed by functions in this section:

```
library(corpcor)
library(mvtnorm)
```

Helper function to convert a symmetric matrix to a vector of the matrix's unique quantities, lower-triangle (including diagonal), column-wise:

```
v<-function(m){m[lower.tri(m,diag=TRUE)]}
```

Helper function to make a model formula for a regression model for selection analysis, taking a list of trait variable names (`z`) and the name of the fitness variable (`w`). This can be used to generate a correct R.H.S. for a Lande & Arnold OLS regression analysis of selection (makes all the right factors of one half for quadratic terms), and can also be used for setting up GLMs for the scheme presented here for relating parameters of log-quadratic functions to selection gradients. For the latter analysis, this function also is useful for ordering the quadratic and interaction terms in a way that is convenient for other functions provided in this section:

```
make.formula<-function(z,W="W"){
  p<-length(z)
  iInd<-v(matrix(1:p,p,p,byrow=FALSE))
  jInd<-v(matrix(1:p,p,p,byrow=TRUE))

  terms<-array(dim=2*p+(p^2-p)/2)
  terms[1:p]<-z
  for(i in 1:(p+(p^2-p)/2)){
    if(iInd[i]==jInd[i]){
      terms[p+i]<-paste("I(0.5*",z[iInd[i]],"*",z[jInd[i]],")",sep="")
    }else{
      terms[p+i]<-paste("I(",z[iInd[i]],"*",z[jInd[i]],")",sep="")
    }
  }
  terms<-paste(terms,collapse="+")
  paste(W,"~",terms,sep="")
}
```

All functions that follow have a commong scheme for arguments:

- `b`: vector of (log) linear regression coefficients

- `gij`: vector of unique entries in the **g** matrix, arranged sub-diagonall column-wise. This correct ordering can be generated by setting up a model formula in the manner generated by the function `make.formula()`, above
- `mu`: vector of mean phenotype
- `Sigma`: phenotypic variance-covariance matrix

A function to construct the matrix g from vectors containing its diagonal and off-diagonal elements, as obtained from a long-link glm regression model:

```
make.g<-function(gij,p,Sigma,warn=FALSE){
  g<-matrix(NA,p,p)
  g[lower.tri(g,diag=TRUE)]<-gij
  g[upper.tri(g)]<-t(g)[upper.tri(t(g))]
  if(warn){
    if(is.positive.definite(solve(Sigma)-g)==FALSE){
      print("Warning: Omega is not positive-definite.")
    }
  }
  g
}
```

Returns an estimate of $\boldsymbol{\beta}$, given **b**, elements of **g**, and the mean vector and covariance matrix of phenotype (assumes multivariate normal phenotype):

```
beta<-function(b,gij,mu,Sigma){
  p<-length(b)
  g<-make.g(gij=gij,p=p,Sigma=Sigma)
  tildeb<-b+g%*%mu
  Phi<-diag(p)-g%*%Sigma
  solve(Phi)%*%tildeb
}
```

Returns an estimate of $\boldsymbol{\gamma}$, given **b**, elements of **g**, and the mean vector and covariance matrix of phenotype (assumes multivariate normal phenotype):

```
gamma<-function(b,gij,mu,Sigma){
  p<-length(b)
  g<-make.g(gij=gij,p=p,Sigma=Sigma)
  tildeb<-b+g%*%mu
  Phi<-diag(p)-g%*%Sigma
  beta<-solve(Phi)%*%tildeb
  beta %*% t(beta) + solve(Phi)%*% g
}
```

Function to calculate both directional and quadratic selection gradients, as well as to return their standard errors. In addition to the function arguments detailed above, `vcov` specifies the sampling variance-covariance matrix of the `b` and `gij` parameters:

```
grads<-function(b,gij,mu,Sigma,vcov,returnCov=FALSE){

  beta_hat<-beta(b,gij,mu,Sigma)
  gamma_hat<-gamma(b,gij,mu,Sigma)

  p<-length(b)
  s<-p+(p^2-p)/2
  phi<-diag(p)-make.g(gij,p)%*%Sigma
  Q<-solve(phi)
```

```
  iInd<-v(matrix(1:p,p,p,byrow=FALSE))
  jInd<-v(matrix(1:p,p,p,byrow=TRUE))

  J12<-matrix(0,p,s)
  for(m in 1:s){
    e<-matrix(0,s,1); e[m,1]<-1;
    C<-matrix(0,p,p);
    C[iInd[m],jInd[m]]<-1
    C[jInd[m],iInd[m]]<-1
    J12<-J12+Q%*%C%*%(Sigma%*%beta_hat+mu)%*%t(e)
  }

  J21<-matrix(0,s,p)
  for(k in 1:p){
    e<-matrix(0,p,1); e[k,1]<-1;
    J21<-J21+ v(beta_hat%*%t(Q[,k])+Q[,k]%*%t(beta_hat))%*%t(e)
  }

  J22<-matrix(0,s,s)
  for(m in 1:s){
    e<-matrix(0,s,1); e[m,1]<-1;
    C<-matrix(0,k,k);
    C[iInd[m],jInd[m]]<-1
    C[jInd[m],iInd[m]]<-1
    M<-Q%*%C%*%(Sigma%*%beta_hat+mu)%*%t(beta_hat)
    J22<-J22+v(M+t(M)+Q%*%C%*%t(Q))%*%t(e)
  }

  J<-matrix(NA,k+s,k+s)
  J[1:p,1:p]<-Q
  J[1:p,(p+1):(p+s)]<-J12
  J[(p+1):(p+s),1:p]<-J21
  J[(p+1):(p+s),(p+1):(p+s)]<-J22

  SEs<-sqrt(diag(J %*% vcov %*% t(J)))

  coef.names<-c(paste("beta",1:p),paste("gamma",iInd,jInd))
  res<-as.data.frame(cbind(coef.names,
    c(beta_hat,gamma_hat[lower.tri(gamma_hat,diag=TRUE)]),SEs))
  names(res)<-c("selection gradient","estimate","SE")
  res[,2]<-as.numeric(as.character(res[,2]))
  res[,3]<-as.numeric(as.character(res[,3]))
  if(returnCov==TRUE) res<-list(grads=res,vcov=J %*% vcov %*% t(J))
  return(res)
}
```

Example application of log-linear fitness model functions

Some simulated trait and fitness data:

```
n<-300
Sigma<-matrix(c(1,0.5,0,0.5,1,0,0,0,1),3,3)
mu<-rep(0,3)
```

```
z<-rmvnorm(n,mu,Sigma)
W<-rpois(n,exp(0.2*z[,1]))
d<-as.data.frame(cbind(W,z))
names(d)<-c("W","z1","z2","z3")
```

Fit a log-link GLM, making use of teh `as.formula()` function in order to get the quadratic and correlational terms in the order that will be expected by subsequent functions:

```
f<-make.formula(W="W",z=c("z1","z2","z3"))
mod<-glm(as.formula(f),data=d,family="poisson")
```

The coefficeints of the log-link GLM:

```
summary(mod)$coefficients
```

```
##                      Estimate Std. Error     z value     Pr(>|z|)
## (Intercept)      -0.045999143 0.08793219 -0.52312062 0.6008903192
## z1                0.285088133 0.07378211  3.86391963 0.0001115819
## z2                0.034433626 0.07172306  0.48009140 0.6311624051
## z3               -0.065313186 0.06343231 -1.02965168 0.3031735447
## I(0.5 * z1 * z1) -0.036611279 0.11395334 -0.32128307 0.7479958839
## I(z2 * z1)       -0.027952158 0.09440434 -0.29608975 0.7671615382
## I(z3 * z1)       -0.005013283 0.06635161 -0.07555630 0.9397721011
## I(0.5 * z2 * z2)  0.032107894 0.10289403  0.31204816 0.7550039194
## I(z3 * z2)       -0.003552515 0.07277386 -0.04881582 0.9610660770
## I(0.5 * z3 * z3)  0.117135628 0.08697906  1.34671060 0.1780734565
```

```
b<-coef(mod)[2:4]
gij<-coef(mod)[5:10]
```

The error variance-covariance matrix of the `b` and `gij` terms:

```
sampVar<-vcov(mod)[2:10,2:10]
```

The selection gradients and their standard errors:

```
gradients<-grads(b,gij,mu,Sigma,sampVar)
gradients
```

```
##   selection gradient     estimate         SE
## 1             beta 1  0.270312651 0.06652351
## 2             beta 2  0.032069852 0.07014393
## 3             beta 3 -0.076277629 0.06785593
## 4          gamma 1 1  0.039480203 0.11304805
## 5          gamma 2 1 -0.019373514 0.09816367
## 6          gamma 3 1 -0.025846471 0.07522536
## 7          gamma 2 2  0.034083710 0.10757590
## 8          gamma 3 2 -0.006481268 0.08572503
## 9          gamma 3 3  0.138562982 0.11403880
```

A small simulation study of the performance of the log-linear selection gradient estimators, in comparison to existing methods

This function takes an (absolute) fitness measure (`W`) and a univariate trait (`z`) and generates bootstrap standard errors for a univariate Lande & Arnold OLS selection analysis with a directional and quadratic term:

```
LAboot<-function(W,z){
  bs<-array(dim=c(n.boot,2))
  for(i in 1:n.boot){
    boot.ord<-sample(1:length(W),length(W),replace=TRUE)
    bW<-W[boot.ord]
    bz<-z[boot.ord]
    m<-lm(I(bW/mean(bW))~bz+I(0.5*bz^2))
    bs[i,]<-coef(m)[2:3]
  }
  apply(bs,2,sd)
}
```

Core simulation function. Generates a sample of phenotype (mean zero, unit variance), and fitness from a Poisson regression model with directional and quadratic terms:

```
sim.grads<-function(n,b,g,a=0){
  z<-rnorm(n,0,1)
  EW<-exp(a + b*z + g*0.5*z^2)
  W<-rpois(n,EW)
  m1<-lm(I(W/mean(W))~z+I(0.5*z^2))
  m2<-glm(W~z+I(0.5*z^2),family="poisson")
  d<-data.frame(W=W,z=z)
  res<-list()
  res$true_beta<-b/(1-g)
  res$true_gamma<-(b^2+g*(1-g))/((1-g)^2)
  res$LA_beta<-coef(m1)[2]
  res$LA_beta_SE<-summary(m1)$coefficients[2,2]
  res$LA_gamma<-coef(m1)[3]
  res$LA_gamma_SE<-summary(m1)$coefficients[3,2]
  bsSE<-LAboot(W,z)
  res$LA_beta_bootSE<-bsSE[1]
  res$LA_gamma_bootSE<-bsSE[2]
  b<-coef(m2)[2]; g<-coef(m2)[3]
  v<-vcov(m2)[2:3,2:3]
  res$MG_beta<-b/(1-g)
  res$MG_beta_SE<-sqrt(v[1,1]/((1-g)^2)+(b^2*v[2,2])/((1-g)^4)
       +((2*b)*v[1,2])/(1-g)^3 )
  res$MG_gamma<-(b^2+g*(1-g))/((1-g)^2)
  res$MG_gamma_SE<-sqrt( (4*b^2*v[1,1])/((1-g)^4)
      + ((1+2*b^2-g)^2*v[2,2])/((1-g)^6)
      + ((4*b*(1+2*b^2-g))*v[1,2])/(1-g)^5 )
  res
}
```

```
run.sims<-function(n,b,g,n.sims=1000,progress=FALSE){
    r<-as.data.frame(array(dim=c(n.sims,12)))
    names(r)<-c("true_beta","true_gamma","LA_beta",
         "LA_beta_SE","LA_gamma","LA_gamma_SE",
         "LA_beta_bootSE","LA_gamma_bootSE",
         "MG_beta","MG_beta_SE","MG_gamma","MG_gamma_SE")
    for(i in 1:n.sims){
      r[i,]<-unlist(sim.grads(n,b,g))
      if(progress==TRUE & i%%100 == 0) print(paste(round(r[i,1],2),round(r[i,2],2),i))
    }
```

```
    sims.res<-list()
    sims.res$b<-b
    sims.res$g<-g
    sims.res$beta_true<-r$true_beta[1]
    sims.res$gamma_true<-r$true_gamma[1]
    sims.res$LA_beta_bias<-mean(r$LA_beta-r$true_beta)
    sims.res$LA_gamma_bias<-mean(r$LA_gamma-r$true_gamma)
    sims.res$LA_beta_sampSD<-sd(r$LA_beta)
    sims.res$LA_gamma_sampSD<-sd(r$LA_gamma)
    sims.res$LA_beta_meanSE<-mean(r$LA_beta_SE)
    sims.res$LA_gamma_meanSE<-mean(r$LA_gamma_SE)
    sims.res$LA_beta_meanBootSE<-mean(r$LA_beta_bootSE)
    sims.res$LA_gamma_meanBootSE<-mean(r$LA_gamma_bootSE)

    sims.res$LA_beta_MAE<-mean(abs(r$LA_beta-r$true_beta[1]))
    sims.res$LA_gamma_MAE<-mean(abs(r$LA_gamma-r$true_gamma[1]))
    sims.res$LA_beta_MSE<-mean((r$LA_beta-r$true_beta[1])^2)
    sims.res$LA_gamma_MSE<-mean((r$LA_gamma-r$true_gamma[1])^2)


    sims.res$MG_beta_bias<-mean(r$MG_beta-r$true_beta)
    sims.res$MG_gamma_bias<-mean(r$MG_gamma-r$true_gamma)
    sims.res$MG_beta_sampSD<-sd(r$MG_beta)
    sims.res$MG_gamma_sampSD<-sd(r$MG_gamma)
    sims.res$MG_beta_meanSE<-mean(r$MG_beta_SE)
    sims.res$MG_gamma_meanSE<-mean(r$MG_gamma_SE)

    sims.res$MG_beta_MAE<-mean(abs(r$MG_beta-r$true_beta[1]))
    sims.res$MG_gamma_MAE<-mean(abs(r$MG_gamma-r$true_gamma[1]))
    sims.res$MG_beta_MSE<-mean((r$MG_beta-r$true_beta[1])^2)
    sims.res$MG_gamma_MSE<-mean((r$MG_gamma-r$true_gamma[1])^2)

    list(summary=sims.res, full.sims=r)
}
```

```
n.sims<-1000  # simulations for each parameter combination
n.boot<-1000  # number of bottstrap samples for alternate OLS SEs
dens<-11      # number of values of b
n<-200        # simulation sample size
g.vals<-c(-1,0,0.4) # values of g to consider
```

Control variable for brute-force caching, controls whether results are generated or read from a file (see below), and whether results (either generated new or read from a file) are written to a results file.

```
runSims<-FALSE
saveSims<-FALSE
resFile<-"./univariateSims.RData"
```

Simulations with negative curvature of the fitness function:

```
if(runSims){
  b.range<-seq(-0.5,0.5,length.out=dens)
  g.range<-rep(g.vals[1],dens)
  sim.res<-as.data.frame(array(dim=c(dens,26)))
  for(j in 1:dens){
```

```
      t<-run.sims(n=n,b= b.range[j],g=g.range[j],n.sims=n.sims)
      sim.res[j,]<-unlist(t$summary)
      names(sim.res)<-names(t[[1]])
  }
  directional1<-sim.res
}
```

71 Simulations with no curvature of the fitness function (on the log scale):

```
if(runSims){
b.range<-seq(-0.5,0.5,length.out=dens)
g.range<-rep(g.vals[2],dens)
sim.res<-as.data.frame(array(dim=c(dens,26)))
for(j in 1:dens){
    t<-run.sims(n=n,b= b.range[j],g=g.range[j],n.sims=n.sims)
    sim.res[j,]<-unlist(t$summary)
    names(sim.res)<-names(t[[1]])
}

directional2<-sim.res
}
```

72 Simulations with positive curvature of the fitness function

```
if(runSims){
b.range<-seq(-0.5,0.5,length.out=dens)
g.range<-rep(g.vals[3],dens)
sim.res<-as.data.frame(array(dim=c(dens,26)))
for(j in 1:dens){
    t<-run.sims(n=n,b= b.range[j],g=g.range[j],n.sims=n.sims)
    sim.res[j,]<-unlist(t$summary)
    names(sim.res)<-names(t[[1]])
}

directional3<-sim.res
}
```

73 Save simulation results, if desired:

```
if(saveSims){
  save(directional1,directional2,directional3,file=resFile)
}
```

74 Load simulation results, if necessary:

```
if(runSims==FALSE){
  load(resFile)
}
```

75 Figure for manuscript showing simulation results:

```
seYrangebeta<-c(0,0.3)
seYrangebeta1<-c(0,0.02)
seYrangebeta2<-c(0,0.1)
seYrangegamma<-c(0,0.5)
seYrangegamma1<-c(0,0.1)
seYrangegamma2<-c(0,0.6)
betaBiasYrange<-c(-0.65,0.65)
```

```
make.beta.axes<-function(g,ax.cex=0.6){
    ax.b.vals<-seq(-0.5,0.5,by=0.25)
    ax.beta.vals<-apply(matrix(ax.b.vals,length(ax.b.vals),1),1,
                        function(x){beta(x,g,0,1)})
    axis(side=1,line=0,at= ax.b.vals, ax.b.vals,cex=ax.cex)
    mtext(side=1,line=2,outer=FALSE,"b",cex=ax.cex)
    axis(side=1,line=3,at= ax.b.vals, round(ax.beta.vals,2),cex=ax.cex)
    mtext(side=1,line=5.5,outer=FALSE,expression(beta),cex=ax.cex)
}

make.gamma.axes<-function(g,ax.cex=0.6){
    ax.b.vals<-seq(-0.5,0.5,by=0.25)
    ax.gamma.vals<-apply(matrix(ax.b.vals,length(ax.b.vals),1),1,
                         function(x){gamma(x,g,0,1)})
    axis(side=1,line=0,at= ax.b.vals, ax.b.vals,cex=ax.cex)
    mtext(side=1,line=2,outer=FALSE,"b",cex=ax.cex)
    axis(side=1,line=3,at= ax.b.vals, round(ax.gamma.vals,2),cex=ax.cex)
    mtext(side=1,line=5.5,outer=FALSE,expression(gamma),cex=ax.cex)
}

x<-c(1,2,3,1,2,3,1,2,3,4,5,6,4,5,6,4,5,6)
m<-matrix(c(
x,
0,0,0,
x+6,
0,0,0,
x+12
),20,3,byrow=TRUE)
layout(m)

letter<-1
mk.label<-function(){
  mtext(side=3,outer=FALSE,paste(" (",letters[letter],")",sep=""),
           adj=0,line=-1.2,cex=0.8)
  letter<<-letter+1
}

exp1<-expression(paste("mean of ",hat(beta)))
exp2<-expression(paste("mean SE or SD of ",hat(beta),"    "))
exp3<-expression(paste("MSE of ",hat(beta)))
exp4<-expression(paste("mean of ",hat(gamma)))
exp5<-expression(paste("mean SE or SD of ",hat(gamma),"    "))
exp6<-expression(paste("MSE of ",hat(gamma)))

par(mar=c(6,4.5,0.8,0.8),oma=c(2,2,2,1),las=1)

d<-directional1

plot(d$b,d$beta_true,type="l",col="gray",lwd=3,lty="dashed",
          ylim=betaBiasYrange,xaxt='n',xlab='',ylab=exp1)
lines(d$b,d$beta_true+d$LA_beta_bias,col="red")
lines(d$b,d$beta_true+d$MG_beta_bias,col="blue")
mk.label()
```

```
legend("bottomright",col=c("gray","blue","red"),
       lty=c("dashed","solid","solid"),lwd=c(3,1,1),
       c("true value","log-linear estimate","OLS estimate"),cex=0.7,bty='n')

make.beta.axes(g=g.vals[1])

plot(d$b,d$LA_beta_sampSD,type="l",col="red",ylim= seYrangebeta,
          xaxt='n',xlab='',ylab=exp2)
lines(d$b,d$MG_beta_sampSD,col="blue")
lines(d$b,d$LA_beta_meanSE,col="red",lty="dashed")
lines(d$b,d$LA_beta_meanBootSE,col="red",lty="dotted")
lines(d$b,d$MG_beta_meanSE,col="blue",lty="dashed")
mk.label()
make.beta.axes(g=g.vals[1])
mtext(side=3,outer=FALSE,line=1,"negative curvature (g = -1)")

legend("topright",lty=c("solid"),col=c("blue","red"),
       c("log-linear estimates","OLS estimates"),cex=0.7,bty='n')

plot(d$b,d$LA_beta_MSE,type="l",col="red",ylim= seYrangebeta1,
          xaxt='n',xlab='',ylab=exp3)
lines(d$b,d$MG_beta_MSE,col="blue")
mk.label()

legend("topright",lty=c("solid"),col=c("blue","red"),
       c("log-linear estimates","OLS estimates"),cex=0.7,bty='n')

make.beta.axes(g=g.vals[1])

plot(d$b,d$gamma_true,type="l",col="gray",lwd=3,lty="dashed",
          ylim=c(-0.6,0),xaxt='n',xlab='',ylab=exp4)
lines(d$b,d$gamma_true+d$LA_gamma_bias,col="red")
lines(d$b,d$gamma_true+d$MG_gamma_bias,col="blue")
mk.label()

make.gamma.axes(g=g.vals[1])

plot(d$b,d$LA_gamma_sampSD,type="l",col="red",ylim= seYrangegamma,
          xaxt='n',xlab='',ylab=exp5)
lines(d$b,d$MG_gamma_sampSD,col="blue")
lines(d$b,d$LA_gamma_meanSE,col="red",lty="dashed")
lines(d$b,d$LA_gamma_meanBootSE,col="red",lty="dotted")
lines(d$b,d$MG_gamma_meanSE,col="blue",lty="dashed")
mk.label()

legend("topright",lty=c("solid","dashed","dotted"),
       c("empirical SD","mean SE","mean bootstrap SE"),cex=0.7,bty='n')

make.gamma.axes(g=g.vals[1])

plot(d$b,d$LA_gamma_MSE,type="l",col="red",ylim= seYrangegamma1,
          xaxt='n',xlab='',ylab=exp6)
lines(d$b,d$MG_gamma_MSE,col="blue")
```

```
mk.label()

make.gamma.axes(g=g.vals[1])

d<-directional2

plot(d$b,d$beta_true,type="l",col="gray",lwd=3,lty="dashed",
          ylim=betaBiasYrange,xaxt='n',xlab='',ylab=exp1)
lines(d$b,d$beta_true+d$LA_beta_bias,col="red")
lines(d$b,d$beta_true+d$MG_beta_bias,col="blue")
make.beta.axes(g=g.vals[2])
mk.label()

plot(d$b,d$LA_beta_sampSD,type="l",col="red",ylim= seYrangebeta,xaxt='n',
          xlab='',ylab=exp2)
lines(d$b,d$MG_beta_sampSD,col="blue")
lines(d$b,d$LA_beta_meanSE,col="red",lty="dashed")
lines(d$b,d$LA_beta_meanBootSE,col="red",lty="dotted")
lines(d$b,d$MG_beta_meanSE,col="blue",lty="dashed")
mtext(side=3,outer=FALSE,line=1,"log-linear (g = 0)")
make.beta.axes(g=g.vals[2])
mk.label()

plot(d$b,d$LA_beta_MSE,type="l",col="red",ylim= seYrangebeta1,xaxt='n',
          xlab='',ylab=exp3)
lines(d$b,d$MG_beta_MSE,col="blue")
make.beta.axes(g=g.vals[2])
mk.label()

plot(d$b,d$gamma_true,type="l",col="gray",lwd=3,lty="dashed",ylim=c(-0.2,0.5),
          xaxt='n',xlab='',ylab=exp4)
lines(d$b,d$gamma_true+d$LA_gamma_bias,col="red")
lines(d$b,d$gamma_true+d$MG_gamma_bias,col="blue")
make.gamma.axes(g=g.vals[2])
mk.label()

plot(d$b,d$LA_gamma_sampSD,type="l",col="red",ylim= seYrangegamma,
          xaxt='n',xlab='',ylab=exp5)
lines(d$b,d$MG_gamma_sampSD,col="blue")
lines(d$b,d$LA_gamma_meanSE,col="red",lty="dashed")
lines(d$b,d$LA_gamma_meanBootSE,col="red",lty="dotted")
lines(d$b,d$MG_gamma_meanSE,col="blue",lty="dashed")
make.gamma.axes(g=g.vals[2])
mk.label()

plot(d$b,d$LA_gamma_MSE,type="l",col="red",ylim= seYrangegamma1,
          xaxt='n',xlab='',ylab=exp6)
lines(d$b,d$MG_gamma_MSE,col="blue")
make.gamma.axes(g=g.vals[2])
mk.label()

d<-directional3
```

```
ax.b.vals<-seq(-0.5,0.5,by=0.25)
ax.beta.vals<-apply(matrix(ax.b.vals,length(ax.b.vals),1),1,
                    function(x){beta(x,0.5,0,1)})

plot(d$b,d$beta_true,type="l",col="gray",lwd=3,lty="dashed",
           ylim=betaBiasYrange,xaxt='n',xlab='',ylab=exp1)
lines(d$b,d$beta_true+d$LA_beta_bias,col="red")
lines(d$b,d$beta_true+d$MG_beta_bias,col="blue")
make.beta.axes(g=g.vals[3])
mk.label()

plot(d$b,d$LA_beta_sampSD,type="l",col="red",ylim= seYrangebeta,
          xaxt='n',xlab='',ylab=exp2)
lines(d$b,d$MG_beta_sampSD,col="blue")
lines(d$b,d$LA_beta_meanSE,col="red",lty="dashed")
lines(d$b,d$LA_beta_meanBootSE,col="red",lty="dotted")
lines(d$b,d$MG_beta_meanSE,col="blue",lty="dashed")
mtext(side=3,outer=FALSE,line=1,"positive curvature (g = +0.4)")
make.beta.axes(g=g.vals[3])
mk.label()

plot(d$b,d$LA_beta_MSE,type="l",col="red",ylim= seYrangebeta2,
          xaxt='n',xlab='',ylab=exp3)
lines(d$b,d$MG_beta_MSE,col="blue")
make.beta.axes(g=g.vals[3])
mk.label()

plot(d$b,d$gamma_true,type="l",col="gray",lwd=3,lty="dashed",
          ylim=c(0,1.5),xaxt='n',xlab='',ylab=exp4)
lines(d$b,d$gamma_true+d$LA_gamma_bias,col="red")
lines(d$b,d$gamma_true+d$MG_gamma_bias,col="blue")
make.gamma.axes(g=g.vals[3])
mk.label()

plot(d$b,d$LA_gamma_sampSD,type="l",col="red",ylim= seYrangegamma,
          xaxt='n',xlab='',ylab=exp5)
lines(d$b,d$MG_gamma_sampSD,col="blue")
lines(d$b,d$LA_gamma_meanSE,col="red",lty="dashed")
lines(d$b,d$LA_gamma_meanBootSE,col="red",lty="dotted")
lines(d$b,d$MG_gamma_meanSE,col="blue",lty="dashed")
make.gamma.axes(g=g.vals[3])
mk.label()

plot(d$b,d$LA_gamma_MSE,type="l",col="red",ylim= seYrangegamma2,
          xaxt='n',xlab='',ylab=exp6)
lines(d$b,d$MG_gamma_MSE,col="blue")
make.gamma.axes(g=g.vals[3])
mk.label()
```

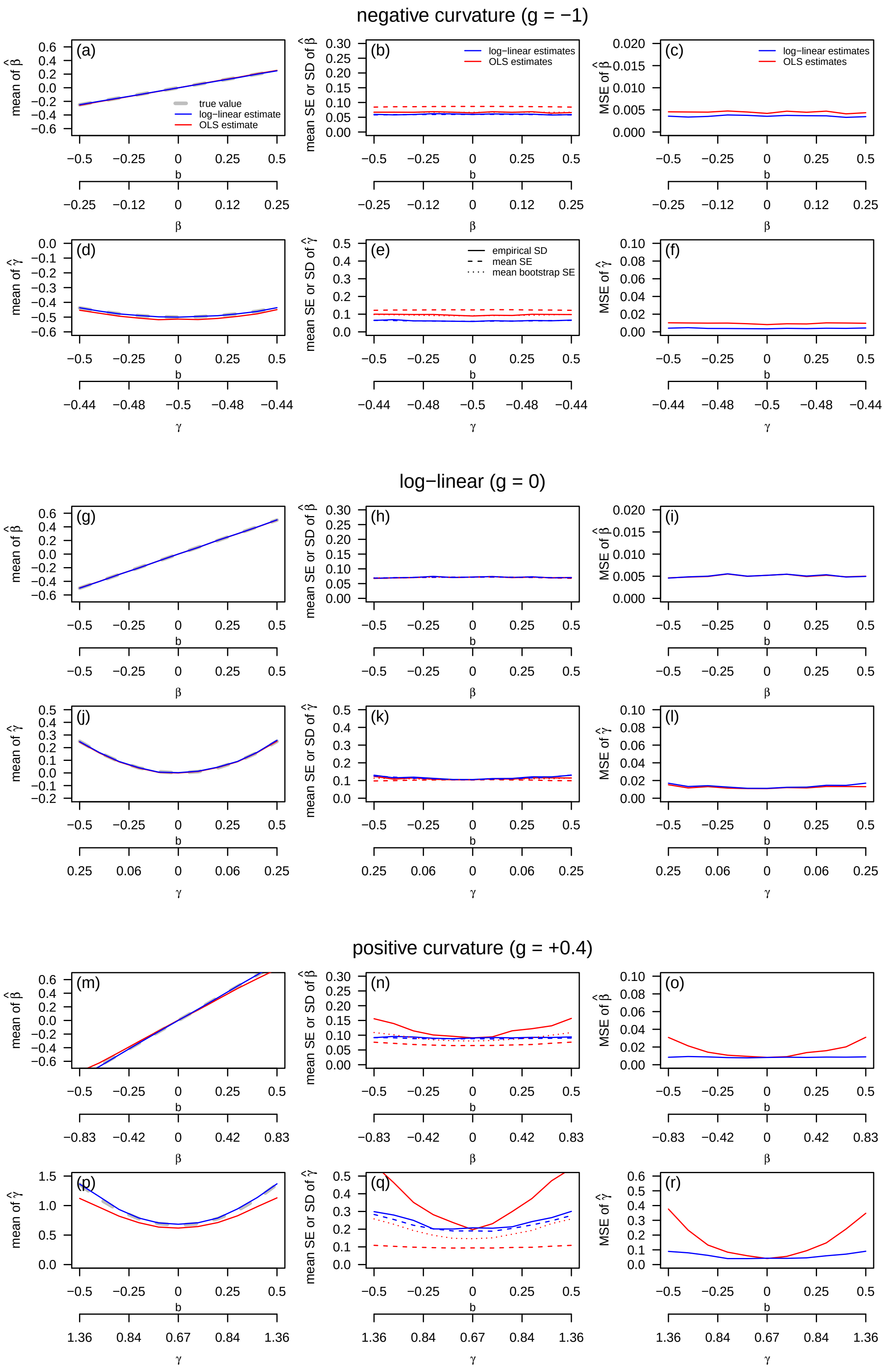
negative curvature (g = −1)
(a)
true value
log−linear estimate
OLS estimate
(b)
log−linear estimates
OLS estimates
(c)
log−linear estimates
OLS estimates
(d)
(e)
empirical SD
mean SE
mean bootstrap SE
(f)
log−linear (g = 0)
(g)
(h)
(i)
(j)
(k)
(l)
positive curvature (g = +0.4)
(m)
(n)
(o)
(p)
(q)
(r)
mean of β̂
mean SE or SD of β̂
MSE of β̂
mean of γ̂
mean SE or SD of γ̂
MSE of γ̂
b
β
γ

Inference of variation in selection in Soay sheep

We will use `MCMCglmm()` to fit the random regression GLMM with additive overdisperion of Poisson errors:

```
library(MCMCglmm)
```

```
## Loading required package: Matrix
## Loading required package: coda
## Loading required package: ape
```

The data (individual ID, birth year, birth mass in kg, and lifetime breeding success):

```
EweLBSData<-read.table("./EweLBSData.csv",sep=',',header=TRUE)
head(EweLBSData)
```

```
##     ID BirthYear BirthMass LBS
## 1 1354      1985      2.14   0
## 2 1355      1985      1.90   0
## 3 1356      1985      1.70   0
## 4 1357      1985      2.44   0
## 5 1358      1985      1.30   0
## 6 1359      1985      1.70   0
```

Generate standardised (zero-centered and unit variance) phenotype records by cohort, plus squared and halved covariate for quadratic regression terms:

```
# cohort means and SDs
cohortMean<-tapply(EweLBSData$BirthMass, EweLBSData$BirthYear,mean)
cohortSD<-tapply(EweLBSData$BirthMass, EweLBSData$BirthYear,sd)

# attach cohort means and SDs to dataset
EweLBSData$cohortMean<-cohortMean[match(EweLBSData$BirthYear,names(cohortMean))]
EweLBSData$cohortSD<-cohortSD[match(EweLBSData$BirthYear,names(cohortSD))]

# standardised directional and quadratic covariates
EweLBSData$sBMass<-(EweLBSData$BirthMass-EweLBSData$cohortMean)/EweLBSData$cohortSD
EweLBSData$halfsBMassSquared<-0.5*EweLBSData$sBMass^2
```

More brute-force caching that allows objects to be explicitly saved and re-loaded:

```
runMainSoayModel<-FALSE
saveMainSoayModel<-FALSE
mainSoayModelFileName<-"mainSoayModel.RData"
```

This is the model in MCMCglmm with random slope and curvature terms. Informative priors are used for the overdispersion variance (see below for more on sensitivity to priors for overdispersion variances), that give relatively low prior probability to very large residual variances:

```
EweLBSData$fbyear<-factor(EweLBSData$BirthYear)

if(runMainSoayModel){
  p<-list(G=list(G1=list(V=diag(2),alpha.mu=rep(0,2),alpha.V=diag(2)*1000,nu=1)),
    R=list(V=diag(length(unique(EweLBSData$BirthYear)))*0.1,nu=10))
  m<-MCMCglmm(LBS~-1+sBMass+halfsBMassSquared+fbyear,
    random=~us(sBMass+ halfsBMassSquared):fbyear,
    nitt=13000*50,thin=10*50,burnin=3000*50,pr=TRUE,
    rcov=~idh(fbyear):units,family="poisson",data= EweLBSData,prior=p,verbose=FALSE)
}else{
```

```
  load(mainSoayModelFileName)
}
if(saveMainSoayModel){
  save(m,file=mainSoayModelFileName)
}
```

Estimates from the random quadratic regression GLMM:

```
summary(m)
```

```
##
##  Iterations = 150001:649501
##  Thinning interval  = 500
##  Sample size  = 1000
##
##  DIC: 3629.398
##
##  G-structure:  ~us(sBMass + halfsBMassSquared):fbyear
##
##                                            post.mean l-95% CI u-95% CI eff.samp
## sBMass:sBMass.fbyear                           0.2511  0.04913  0.51823     1000
## halfsBMassSquared:sBMass.fbyear               -0.2560 -0.55507 -0.02293     1000
## sBMass:halfsBMassSquared.fbyear               -0.2560 -0.55507 -0.02293     1000
## halfsBMassSquared:halfsBMassSquared.fbyear     0.4793  0.08347  1.06613     1000
##
##  R-structure:  ~idh(fbyear):units
##
##                  post.mean l-95% CI u-95% CI eff.samp
## fbyear1985.units    6.8213   2.4921   13.623   1182.6
## fbyear1986.units    0.8708   0.3222    1.570   1000.0
## fbyear1987.units    1.4671   0.7896    2.302   1000.0
## fbyear1988.units    5.6675   2.0925   10.427    872.2
## fbyear1989.units    1.3780   0.5582    2.355   1000.0
## fbyear1990.units    0.9390   0.3842    1.514   1000.0
## fbyear1991.units    2.3169   0.9944    4.138   1000.0
## fbyear1992.units    2.1389   0.9514    3.515   1000.0
## fbyear1993.units    3.1393   1.7351    5.413   1000.0
## fbyear1994.units    4.4548   1.9304    8.143   1000.0
## fbyear1995.units    1.8907   1.0095    2.906    652.2
## fbyear1996.units    4.3600   1.7563    7.517   1000.0
## fbyear1997.units    5.0152   2.0872    8.713   1179.5
## fbyear1998.units    6.6934   2.3814   12.590   1000.0
## fbyear1999.units    0.9051   0.3610    1.533   1110.3
## fbyear2000.units    1.9212   1.0418    2.965   1000.0
## fbyear2002.units    1.0979   0.3695    1.932   1000.0
## fbyear2003.units    1.3494   0.6996    2.073   1000.0
## fbyear2004.units    5.6314   1.7692   11.932   1000.0
## fbyear2005.units    3.5053   1.4275    5.874   1000.0
## fbyear2006.units    2.6934   1.1664    4.490   1018.3
##
##  Location effects: LBS ~ -1 + sBMass + halfsBMassSquared + fbyear
##
##                    post.mean l-95% CI u-95% CI eff.samp  pMCMC
## sBMass               1.06741  0.77263  1.32791    802.1 <0.001 ***
```

```
## halfsBMassSquared  -0.73059 -1.10066 -0.31499   1000.0  0.002 **
## fbyear1985         -4.56116 -6.66290 -2.86973   1000.0 <0.001 ***
## fbyear1986          0.30523 -0.18221  0.77263   1145.2  0.242
## fbyear1987          0.24325 -0.22333  0.71597   1000.0  0.292
## fbyear1988         -3.18997 -4.77639 -2.00678   1000.0 <0.001 ***
## fbyear1989          0.72845  0.13537  1.27530    887.4  0.022 *
## fbyear1990          1.20823  0.81572  1.63137   1000.0 <0.001 ***
## fbyear1991         -0.80561 -1.51423 -0.01222   1000.0  0.016 *
## fbyear1992          0.29842 -0.28860  0.86383   1000.0  0.308
## fbyear1993         -0.28853 -0.96900  0.30351   1000.0  0.424
## fbyear1994         -2.48231 -3.60360 -1.45146   1000.0 <0.001 ***
## fbyear1995          0.40586 -0.11091  0.90628   1000.0  0.138
## fbyear1996         -1.34279 -2.33521 -0.33496   1096.4  0.002 **
## fbyear1997         -1.74549 -2.84380 -0.78042   1094.2  0.002 **
## fbyear1998         -3.54096 -5.16439 -1.67338   1000.0 <0.001 ***
## fbyear1999          0.70576  0.29285  1.10268   1000.0  0.004 **
## fbyear2000          0.22030 -0.27371  0.79796   1000.0  0.408
## fbyear2002          0.48493 -0.08239  0.98375   1000.0  0.116
## fbyear2003          0.82510  0.38484  1.24982   1000.0  0.002 **
## fbyear2004         -3.94018 -6.10490 -2.12844   1000.0 <0.001 ***
## fbyear2005         -0.51893 -1.36574  0.24099   1000.0  0.168
## fbyear2006         -0.70289 -1.35190  0.09354   1000.0  0.036 *
## ---
## Signif. codes:  0 '***' 0.001 '**' 0.01 '*' 0.05 '.' 0.1 ' ' 1
```

Overall selection gradients and their standard errors (selection gradients in an average year), using the linear approximation for the standard errors:

```
grads(b=mean(m$Sol[,1]),gij=mean(m$Sol[,2]),mu=0,Sigma=1,vcov=cov(m$Sol[,2:3]))
```

```
##   selection gradient    estimate        SE
## 1             beta 1  0.61678984 0.3900022
## 2          gamma 1 1 -0.04173389 0.8274124
```

Here is the integration over the posterior distribution of the fitted model that yields the overall estimates, plus inference of the variability of selection among cohorts. Note that what is passed to the `grads()` function is not a descriptor of statistical uncertainty. Rather, it is posterior samples of the among-cohort variance in the **b** and **g** terms:

```
postGradsDistData<-as.data.frame(array(dim=c(1000,5)))
names(postGradsDistData)<-c("beta","gamma","SDBeta","SDGamma","CorBetaGamma")
for(i in 1:1000){
  vcv<-matrix(m$VCV[i,1:4],2,2)
  r<-grads(m$Sol[i,1],m$Sol[i,2],0,1,vcv,returnCov=TRUE)
  postGradsDistData[i,1:2]<-r$grads[,2]
  postGradsDistData[i,3:4]<-r$grads[,3]
  postGradsDistData[i,5]<-cov2cor(r$vcov)[1,2]
}
```

Posterior mean and credible intervals of selection gradients in an average year:

```
apply(postGradsDistData[,1:2],2,mean)
```

```
##        beta       gamma
##  0.61869785 -0.02700945
```

```
apply(postGradsDistData[,1:2],2,function(x){HPDinterval(as.mcmc(x))})
```

```
##            beta      gamma
## [1,] 0.4926804 -0.2740374
## [2,] 0.7348181  0.1906765
```

Posterior mean and credible intervals of SDs and correlation of β and γ across cohorts:

```
apply(postGradsDistData[,3:5],2,mean)
```

```
##       SDBeta      SDGamma CorBetaGamma
##    0.1868023    0.3498531    0.6619660
```

```
apply(postGradsDistData[,3:5],2,function(x){HPDinterval(as.mcmc(x))})
```

```
##          SDBeta    SDGamma CorBetaGamma
## [1,] 0.03996036 0.05531846  -0.08172792
## [2,] 0.34712014 0.68612124   0.99449372
```

Do OLS regressions to help with the graphical illustration of the regression models. These are the same as the regressions for selection analyses, except the response is absolute fitness, not relative fitness, as it would have to be to get β and γ via OLS regression analysis:

```
years<-unique(EweLBSData$BirthYear)

OLSgrads<-as.data.frame(array(dim=c(length(years),6)))
names(OLSgrads)<-c("year","barW","beta","SEbeta","gamma","SEgamma")
OLSgradsModelList<-list()
OLSgrads[,1]<-years

for(i in 1:length(years)){
    s<-subset(EweLBSData, EweLBSData$BirthYear==years[i])
    OLSgrads[i,2]<-mean(s$LBS)
    m_LA<-lm(I(LBS/mean(LBS))~sBMass+halfsBMassSquared,data=s)
    OLSgradsModelList[[i]]<-m_LA
    OLSgrads[i,3:4]<-summary(m_LA)$coefficients[2,1:2]
    OLSgrads[i,5:6]<-summary(m_LA)$coefficients[3,1:2]
}
```

Helper function to plot annual fitness functions from the GLMM model:

```
plot.glmm<-function(year,m){
    x<-seq(-3,3,length.out=50)
    preds.mcmc<-array(dim=c(1000,50))
    alpha<-m$Sol[,paste("fbyear", year,sep="")]
    beta_j<-m$Sol[,"sBMass"]+m$Sol[,paste("sBMass.fbyear.", year,sep="")]
    gamma_j<-m$Sol[,"halfsBMassSquared"]+m$Sol[,paste("halfsBMassSquared.fbyear.",
                                                year,sep="")]
    sigma2<-m$VCV[,paste("fbyear",year,".units",sep="")]
    lines(x,exp(mean(alpha)+mean(sigma2)/2+mean(beta_j)*x+0.5*mean(gamma_j)*x^2),
                                             col="blue")

    for(i in 1:1000){
        preds.mcmc[i,]<-exp(alpha[i]+sigma2[i]/2+beta_j[i]*x+0.5*gamma_j[i]*x^2)
    }
    lims<-array(dim=c(50,2))
    for(j in 1:50){
        lims[j,]<-HPDinterval(as.mcmc(preds.mcmc[,j]))
    }
    lines(x,lims[,1],col="blue",lty="dashed")
```

```
    lines(x,lims[,2],col="blue",lty="dashed")
}
```

Helper function to plot OLS-based quadratic approximations to annual fitness functions:

```
plot.ols<-function(year){
    i<-which(years%in%year)
    x<-seq(-3,3,length.out=50)
    p<-predict(OLSgradsModelList[[i]],newdata=data.frame(sBMass=x,
         halfsBMassSquared=0.5*x^2),se.fit=TRUE)
    lines(x, OLSgrads[i,2]*(p$fit),col="red")
    lines(x,OLSgrads[i,2]*(p$fit+1.96*p$se.fit),col="red",lty="dashed")
    lines(x,OLSgrads[i,2]*(p$fit-1.96*p$se.fit),col="red",lty="dashed")
}
```

Empirical example plot of annual fitness functions:

```
par(mfrow=c(7,3),oma=c(4,4,1,1),mar=c(1,1,1,1),las=1)

letter<-1
mk.label<-function(year){
  mtext(side=3,outer=FALSE,paste(" (",letters[letter],") ",year,sep=""),
                      adj=0,line=-1.2,cex=0.8)
  letter<<-letter+1
}

for(y in years){
    s<-subset(EweLBSData, EweLBSData$BirthYear==y)
    plot(s$sBMass,s$LBS,ylim=c(0,21),col="gray",xlim=c(-2.4,2.4),xaxt='n',yaxt='n')

    if(letter==1) legend(-0.5,20,col=c("blue","red"),lty="solid",
                         c("log-linear model","OLS"),bty='n',cex=0.85)

    if(letter %% 3 == 1){
      axis(side=2,at=seq(0,20,by=5),seq(0,20,by=5))
    }else{
      axis(side=2,at=seq(0,20,by=5),rep("",5))
    }
    if(letter > (3*6) ){
      axis(side=1,at=-2:2,-2:2)
    }else{
      axis(side=1,at=-2:2,rep("",5))
    }
    mk.label(y)
    plot.glmm(y,m)
    plot.ols(y)
}

mtext(side=1,outer=TRUE,"phenotype (birth mass, variance standardised)",line=2)
mtext(side=2,outer=TRUE,"absolute fitness (lifetime breeding success)",las=0,line=2)
```

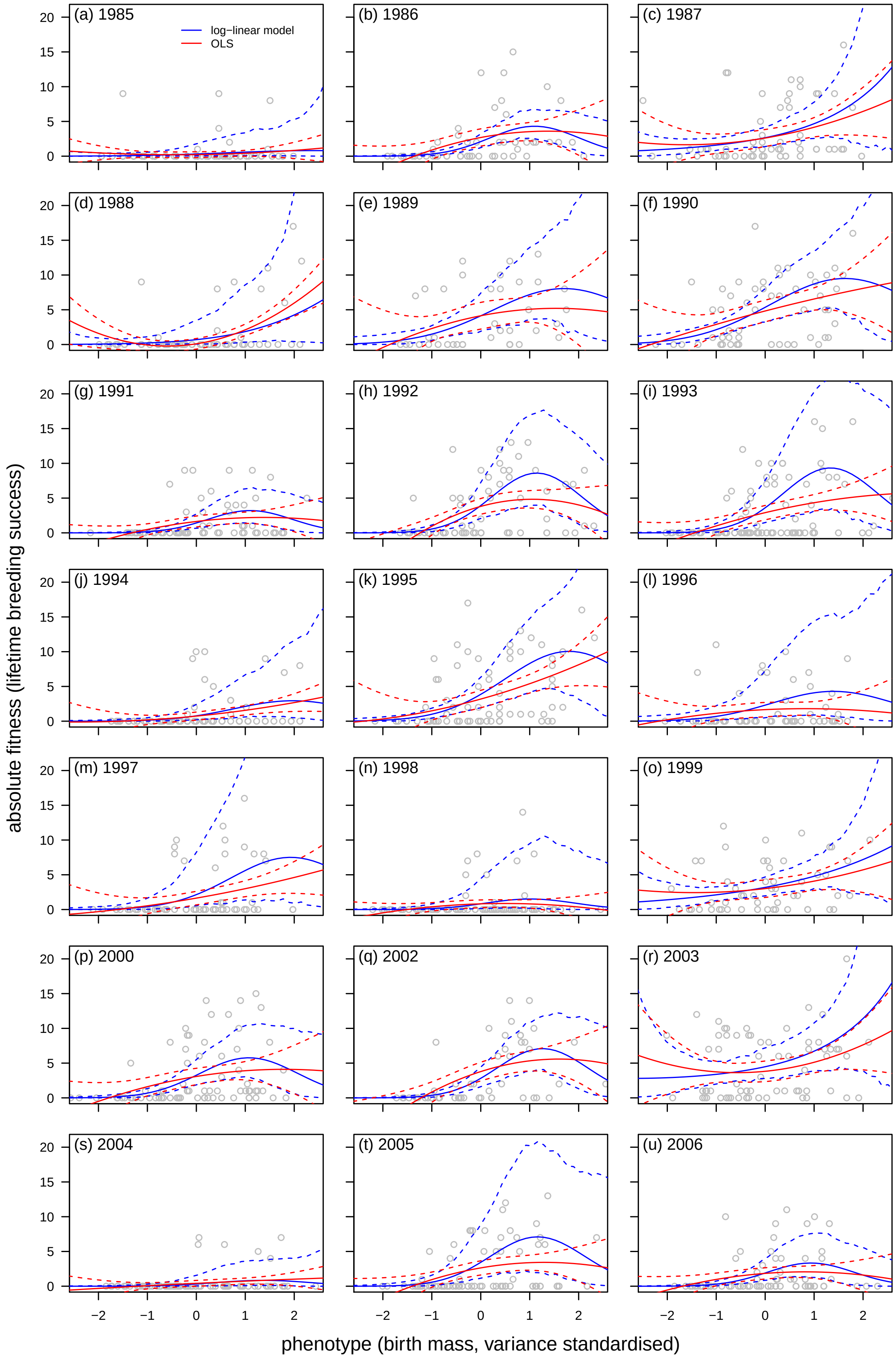
(a) 1985
log−linear model
OLS
(b) 1986
(c) 1987
(d) 1988
(e) 1989
(f) 1990
(g) 1991
(h) 1992
(i) 1993
(j) 1994
(k) 1995
(l) 1996
(m) 1997
(n) 1998
(o) 1999
(p) 2000
(q) 2002
(r) 2003
(s) 2004
(t) 2005
(u) 2006
absolute fitness (lifetime breeding success)
phenotype (birth mass, variance standardised)

Inference of variation in selection with different priors

More brute-force caching for the alternate Soay model:

```
runOtherSoayModels<-FALSE
saveOtherSoayModels<-FALSE
otherSoayModelsFileName<-"otherSoayModels.RData"
```

If more typical priors, corresponding to $gamma(0.001, 0.001)$ for the precision, are used for the residual variance

```
if(runOtherSoayModels){
  p2<-list(G=list(G1=list(V=diag(2),alpha.mu=rep(0,2),alpha.V=diag(2)*1000,nu=1)),
    R=list(V=diag(length(unique(EweLBSData$BirthYear))),nu=0.002))
  m2<-MCMCglmm(LBS~-1+sBMass+halfsBMassSquared+fbyear,
    random=~us(sBMass+ halfsBMassSquared):fbyear,
    nitt=13000*50,thin=10*50,burnin=3000*50,pr=TRUE,
    rcov=~idh(fbyear):units,family="poisson",data= EweLBSData,prior=p2,verbose=FALSE)

  p3<-list(G=list(G1=list(V=diag(2),alpha.mu=rep(0,2),alpha.V=diag(2)*1000,nu=1)),
    R=list(V=1,nu=0.002))
  m3<-MCMCglmm(LBS~-1+sBMass+halfsBMassSquared+fbyear,
    random=~us(sBMass+ halfsBMassSquared):fbyear,
    nitt=13000*50,thin=10*50,burnin=3000*50,pr=TRUE,
    rcov=~units,family="poisson",data= EweLBSData,prior=p3,verbose=FALSE)

  }else{
  load(otherSoayModelsFileName)
}
if(saveOtherSoayModels){
  save(m2,m3,file=otherSoayModelsFileName)
}
```

Here is figure 2, except without the informative priors to control the overdispersion variances:

```
par(mfrow=c(7,3),oma=c(4,4,1,1),mar=c(1,1,1,1),las=1)

letter<-1
mk.label<-function(year){
  mtext(side=3,outer=FALSE,paste(" (",letters[letter],") ",year,sep=""),
                         adj=0,line=-1.2,cex=0.8)
  letter<<-letter+1
}

for(y in years){
    s<-subset(EweLBSData, EweLBSData$BirthYear==y)
    plot(s$sBMass,s$LBS,ylim=c(0,21),col="gray",xlim=c(-2.4,2.4),xaxt='n',yaxt='n')

    if(letter==1) legend(-0.5,20,col=c("blue","red"),lty="solid",
                         c("log-linear model","OLS"),bty='n',cex=0.85)

    if(letter %% 3 == 1){
      axis(side=2,at=seq(0,20,by=5),seq(0,20,by=5))
    }else{
      axis(side=2,at=seq(0,20,by=5),rep("",5))
    }
```

```
    if(letter > (3*6) ){
      axis(side=1,at=-2:2,-2:2)
    }else{
      axis(side=1,at=-2:2,rep("",5))
    }
    mk.label(y)
    plot.glmm(y,m2)
    plot.ols(y)
}

mtext(side=1,outer=TRUE,"phenotype (variance standardised)",line=2)
mtext(side=2,outer=TRUE,"absolute fitness (lifetime breeding success)",las=0,line=2)
```

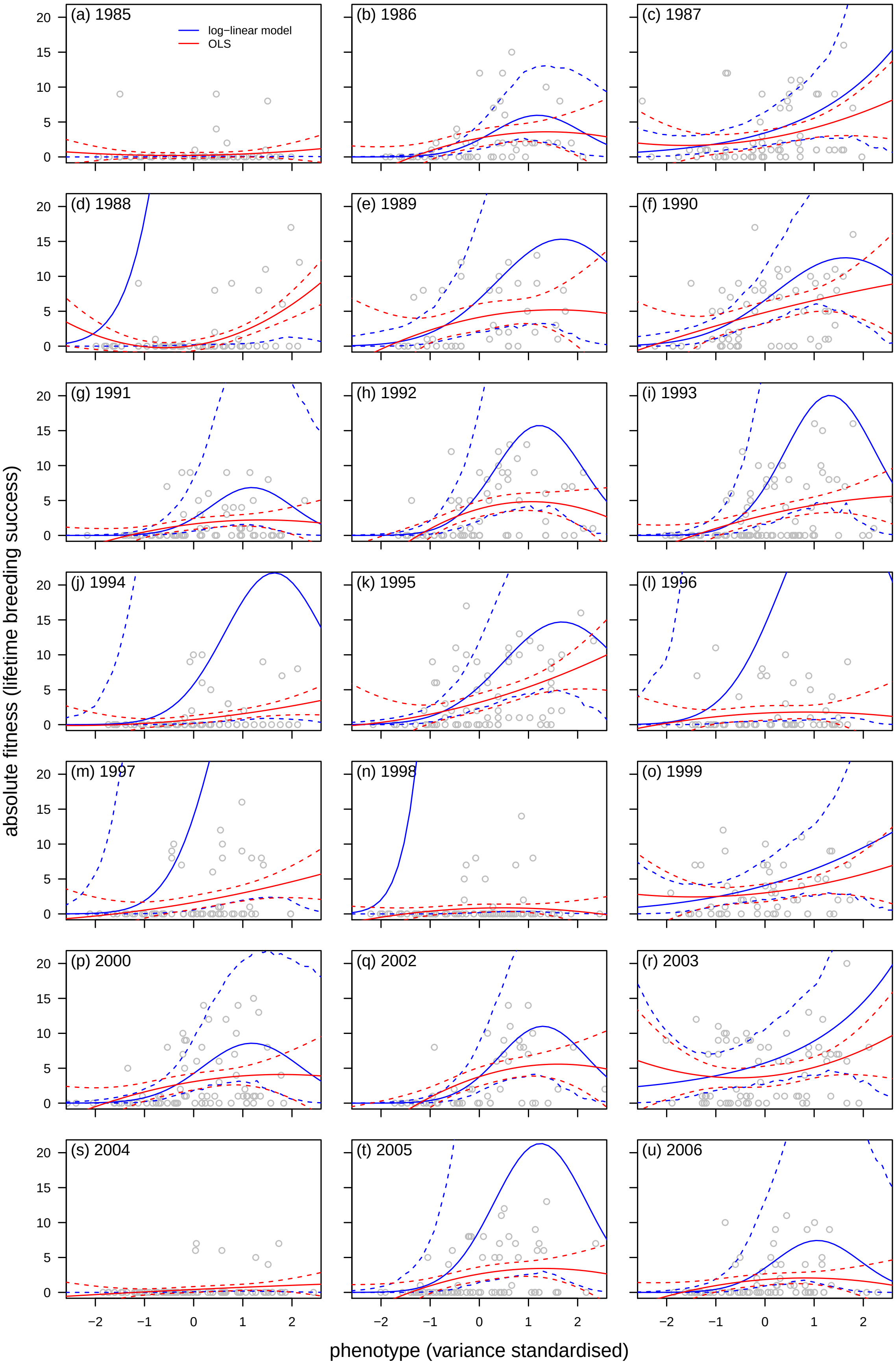
(a) 1985
log−linear model
OLS
(b) 1986
(c) 1987
(d) 1988
(e) 1989
(f) 1990
(g) 1991
(h) 1992
(i) 1993
(j) 1994
(k) 1995
(l) 1996
(m) 1997
(n) 1998
(o) 1999
(p) 2000
(q) 2002
(r) 2003
(s) 2004
(t) 2005
(u) 2006
absolute fitness (lifetime breeding success)
phenotype (variance standardised)

198

Although the model doesn't predict annual fitness very well, the inference of the distribution of selection gradients isn't that much different:

```
postGradsDistData2<-as.data.frame(array(dim=c(1000,5)))
names(postGradsDistData2)<-c("beta","gamma","SDBeta","SDGamma","CorBetaGamma")
for(i in 1:1000){
  vcv<-matrix(m2$VCV[i,1:4],2,2)
  r<-grads(m2$Sol[i,1],m2$Sol[i,2],0,1,vcv,returnCov=TRUE)
  postGradsDistData2[i,1:2]<-r$grads[,2]
  postGradsDistData2[i,3:4]<-r$grads[,3]
  postGradsDistData2[i,5]<-cov2cor(r$vcov)[1,2]
}
```

Posterior mean and credible interval of selection gradients in an average year:

```
apply(postGradsDistData2[,1:2],2,mean)
```

```
##        beta       gamma
## 0.673460326 0.005968415
```

```
apply(postGradsDistData2[,1:2],2,function(x){HPDinterval(as.mcmc(x))})
```

```
##           beta      gamma
## [1,] 0.5422776 -0.2357181
## [2,] 0.8267369  0.2732264
```

Posterior mean and credible interval of SDs and correlation of β and γ across cohorts

```
apply(postGradsDistData2[,3:5],2,mean)
```

```
##       SDBeta      SDGamma CorBetaGamma
##    0.1814336    0.3190772    0.6645310
```

```
apply(postGradsDistData2[,3:5],2,function(x){HPDinterval(as.mcmc(x))})
```

```
##          SDBeta    SDGamma CorBetaGamma
## [1,] 0.02579484 0.03557059   -0.1856045
## [2,] 0.35010080 0.66044499    0.9997540
```

Since the residual structure of m3 is different, it needs a slightly different helper function for plotting:

```
plot.glmm.m3<-function(year){
    x<-seq(-3,3,length.out=50)
    preds.mcmc<-array(dim=c(1000,50))
    alpha<-m3$Sol[,paste("fbyear", year,sep="")]
    beta_j<-m3$Sol[,"sBMass"]+m3$Sol[,paste("sBMass.fbyear.", year,sep="")]
    gamma_j<-m3$Sol[,"halfsBMassSquared"]+m3$Sol[,
                       paste("halfsBMassSquared.fbyear.", year,sep="")]
    sigma2<-m3$VCV[,"units"]
    lines(x,exp(mean(alpha)+mean(sigma2)/2+mean(beta_j)*x+0.5*mean(gamma_j)*x^2),
               col="blue")

    for(i in 1:1000){
        preds.mcmc[i,]<-exp(alpha[i]+sigma2[i]/2+beta_j[i]*x+0.5*gamma_j[i]*x^2)
    }
    lims<-array(dim=c(50,2))
    for(j in 1:50){
        lims[j,]<-HPDinterval(as.mcmc(preds.mcmc[,j]))
    }
```

```
    lines(x,lims[,1],col="blue",lty="dashed")
    lines(x,lims[,2],col="blue",lty="dashed")
}
```

Here is the plot of annual fitness functions, based on the model with the same overdisperion variance in all years:

```
par(mfrow=c(7,3),oma=c(4,4,1,1),mar=c(1,1,1,1),las=1)

letter<-1
mk.label<-function(year){
  mtext(side=3,outer=FALSE,paste(" (",letters[letter],") ",year,sep=""),
        adj=0,line=-1.2,cex=0.8)
  letter<<-letter+1
}

for(y in years){
    s<-subset(EweLBSData, EweLBSData$BirthYear==y)
    plot(s$sBMass,s$LBS,ylim=c(0,21),col="gray",xlim=c(-2.4,2.4),xaxt='n',yaxt='n')

    if(letter==1) legend(-0.5,20,col=c("blue","red"),lty="solid",
                         c("log-linear model","OLS"),bty='n',cex=0.85)

    if(letter %% 3 == 1){
      axis(side=2,at=seq(0,20,by=5),seq(0,20,by=5))
    }else{
      axis(side=2,at=seq(0,20,by=5),rep("",5))
    }
    if(letter > (3*6) ){
      axis(side=1,at=-2:2,-2:2)
    }else{
      axis(side=1,at=-2:2,rep("",5))
    }
    mk.label(y)
    plot.glmm.m3(y)
    plot.ols(y)
}

mtext(side=1,outer=TRUE,"phenotype (variance standardised)",line=2)
mtext(side=2,outer=TRUE,"absolute fitness (lifetime breeding success)",las=0,line=2)
```

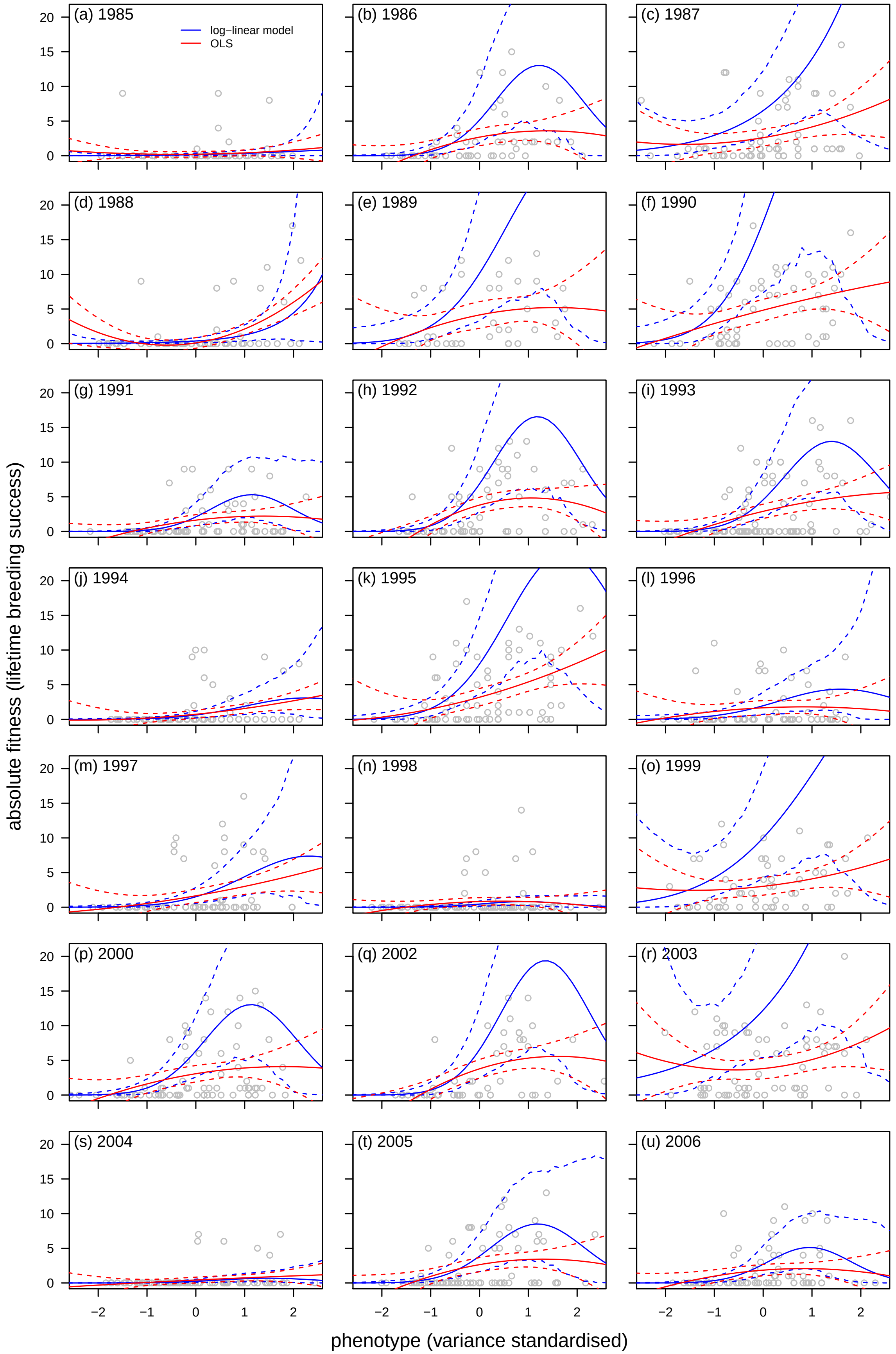
(a) 1985
(b) 1986
(c) 1987
(d) 1988
(e) 1989
(f) 1990
(g) 1991
(h) 1992
(i) 1993
(j) 1994
(k) 1995
(l) 1996
(m) 1997
(n) 1998
(o) 1999
(p) 2000
(q) 2002
(r) 2003
(s) 2004
(t) 2005
(u) 2006
log−linear model
OLS
absolute fitness (lifetime breeding success)
phenotype (variance standardised)

Although this model has poor fit, the gradients are not all that much different:

```
postGradsDistData3<-as.data.frame(array(dim=c(1000,5)))
names(postGradsDistData3)<-c("beta","gamma","SDBeta","SDGamma","CorBetaGamma")
for(i in 1:1000){
  vcv<-matrix(m3$VCV[i,1:4],2,2)
  r<-grads(m3$Sol[i,1],m3$Sol[i,2],0,1,vcv,returnCov=TRUE)
  postGradsDistData3[i,1:2]<-r$grads[,2]
  postGradsDistData3[i,3:4]<-r$grads[,3]
  postGradsDistData3[i,5]<-cov2cor(r$vcov)[1,2]
}
```

Posterior mean and credible interval of selection gradients in an average year (model with a single overdispersion variance):

```
apply(postGradsDistData3[,1:2],2,mean)
```

```
##       beta      gamma
## 0.69303347 0.05916951
```

```
apply(postGradsDistData3[,1:2],2,function(x){HPDinterval(as.mcmc(x))})
```

```
##           beta      gamma
## [1,] 0.5437747 -0.2328393
## [2,] 0.8542453  0.3812428
```

Posterior mean and credible interval of SDs and correlation of β and γ across cohorts (model with a single overdispersion variance):

```
apply(postGradsDistData3[,3:5],2,mean)
```

```
##       SDBeta      SDGamma CorBetaGamma
##    0.2250857    0.4908221    0.8865092
```

```
apply(postGradsDistData3[,3:5],2,function(x){HPDinterval(as.mcmc(x))})
```

```
##          SDBeta   SDGamma CorBetaGamma
## [1,] 0.05679567 0.1041716    0.6567395
## [2,] 0.39809739 0.9169985    0.9997729
```
